# Coaching as a growth- or security-oriented process–How regulatory fit increases coaching success

**DOI:** 10.1371/journal.pone.0286059

**Published:** 2023-10-05

**Authors:** Christina Mühlberger, Andreas Maximilian Böhm, Jochim Hansen, Peter Behrendt, Monika Wastian, Eva Jonas

**Affiliations:** 1 Department of Psychology, University of Salzburg, Salzburg, Austria; 2 Dr. Exler & Dr. Kuptsch, Munich, Germany; 3 Freiburg Institut, Freiburg, Germany; 4 Institut für Organisationspsychologie, Munich, Germany; 5 Institut für Organisationspsychologie, Glasgow, United Kingdom; Mugla Sitki Kocman University: Mugla Sitki Kocman Universitesi, TURKEY

## Abstract

Regulatory focus theory suggests that promoters are more concerned with growth and preventers are more concerned with security. Since coaching is a growth-oriented process, it seems to be more suitable for clients high on promotion than for clients high on prevention. Applying regulatory fit theory, the present research investigates how preventers can also benefit from coaching. First, a study looking at real coaching processes (*N*_1_ = 103) found that a higher promotion than prevention focus was indeed related to more coaching success, i.e., satisfaction and approach motivation. Next, testing the hypothesis that fit effects should also be present in coaching, a study using a vignette approach (*N*_2_ = 99) shows that participants experiencing a fit between their focus and a promotion versus a prevention coaching indicate a better coaching evaluation than participants experiencing no fit. In three studies (*N*_3a_ = 120, *N*_3b_ = 85, *N*_3c_ = 189), we used an experimental approach and manipulated the regulatory focus of coaching interventions. We found promotion as well as prevention fit effects showing that participants experiencing a fit indicate more coaching success than participants experiencing no fit. Two studies (*N*_4a_ = 41, *N*_4b_ = 87) further tested interpersonal fit, i.e., the fit between the coach’s and client’s regulatory focus. We found promotion as well as prevention fit effects on participants’ satisfaction with and trust in a coach (Study 4a) and promotion fit effects on participants’ goal attainment and coaching progress (4b). The findings suggest that by adapting coaching to the client’s focus, coaching success can be increased not only for promoters but also for preventers. Thus, we found that regulatory fit effects, albeit small to medium, are also present in coaching. Multiple studies assessing multiple variables relevant to coaching showed that the findings differ regarding the interventions used and the variables that we looked at. The practical implications of these findings are discussed.

## Introduction

According to regulatory focus theory [1], individuals use two self-regulatory systems to navigate through the environment—the growth-oriented promotion system and the security-oriented prevention system. Individuals can differ in the expression of the promotion and prevention system [1] such that promoters (i.e., individuals who are rather driven by the promotion system) are oriented towards growth, change, and success, and preventers (i.e., individuals who are rather driven by their prevention system) are oriented towards security and the avoidance of risks and failure. Previous research has shown the benefits of an individual’s perceived person-environment fit, in particular a regulatory fit between one’s self-regulatory system and the current situation [2]. A situation that may be more suitable for promoters than for preventers is the coaching situation. Coaching can be seen as a growth- and change-oriented process [3–9]. As promoters are growth- and change-oriented and preventers are security-oriented and rather change-aversive, coaching may in general be more successful when coaching clients are more promotion- than prevention-focused. But can preventers who have difficulties disengaging themselves from their status quo also benefit from coaching? If prevention-oriented clients set goals that match their prevention focus, a typically growth-oriented coaching process that uses promotion-focused interventions would be at odds with the client’s prevention focus. Imagine, for example, a person hiring a coach to eliminate stress factors in their job (i.e., a prevention-focused coaching goal). If the coach chooses promotion-oriented interventions such as developing new relaxation strategies, the client’s motivation to work on their goal may decrease. If the coach chooses prevention-oriented interventions such as exploring existing coping strategies which the client already applied in similar situations, the client’s motivation may increase as the intervention fits the regulatory focus.

In the present research, we first investigate the hypothesis that in general, individuals with a promotion focus benefit more from coaching than individuals with a prevention focus. Second, we apply regulatory fit theory [2] to coaching. Given the extensive research on the positive effects of regulatory fit in different areas, we investigate the hypothesis that the effectiveness of a regulatory fit also exists in coaching. We especially focus on individuals high on prevention who should benefit more from coaching when the coach uses prevention-oriented interventions rather than promotion-oriented interventions. Since there is little literature on regulatory fit in coaching, we do not know how important regulatory fit is for coaching and consequently, which coaching variables are particularly affected by regulatory fit. Thus, the aim of the current article is two-fold, examining whether applying regulatory fit to coaching has benefits and examining the coaching outcomes that are particularly affected by regulatory fit. We chose a multimethod approach (investigating real and imagined coaching processes; paper-pencil and online studies, and a behavioral observation study; assessing regulatory focus and qualitatively analyzing regulatory focus; creating a fit by developing promotion- vs. prevention-oriented coaching descriptions and interventions, and by analyzing the language of coach and client), different samples (real coaching clients, student samples, participants working in different professions), and multiple dependent variables that have shown to be relevant variables for coaching success.

### Coaching and outcome taxonomies

Coaching is a consultancy format which is applied to different contexts and approaches. Research has shown that coaching is successful including, for example, high satisfaction, well-being, and goal attainment [3]. There are various definitions of what coaching is. For example, it is defined as “a human development process (…) to promote desirable and sustainable change for the benefit of the client and potentially other stakeholders” [4 p1], in which “organizationally, professionally, and personally beneficial development goals” [5 p73] are identified and achieved. It is a process which has the purpose of “fostering the on-going self-directed learning and personal growth of the client” [6 p2] and “achieving some type of change, learning or new level of individual or organizational performance” [7 p15]. Other definitions also emphasize the aim of self-change, self-development, learning, and performance [8, 9]. Summarizing these definitions, there seems to be agreement that coaching is an interaction between a coach and a coachee, aimed at the achievement of goals, change, growth, and self-development.

Meta-analyses show positive effects of coaching on many outcome variables [5, 10–15]. In our studies, we use different outcomes and classify them according to different taxonomies: Kirkpatrick [16] provides a taxonomy of training evaluation consisting of the categories reaction (e.g., satisfaction), learning (e.g., declarative knowledge), behavior (e.g., changes in leadership behavior), and result (e.g., organizational performance). Regarding the learning category, Kraiger et al. [17] differs between affective outcomes, i.e. attitudinal and motivational change such as positive affect, cognitive outcomes, i.e., knowledge acquisition or cognitive strategies such as self-awareness or cognitive flexibility, and performance and skill-based learning outcomes (e.g., vocational skills). Four coaching meta-analyses [10, 11, 14, 15] suggested the outcome category “psychological well-being” which aims at longer-term/distal well-being-related constructs (e.g., life satisfaction). Given that goal attainment is a central criterion to measure coaching success [e.g., 18], a review article on goal activities in coaching [19] and two meta-analyses [12, 15] proposed adding a goal category. Moreover, one meta-analysis [5] suggested the category relationship outcomes that includes for example trust, credibility, or the working alliance.

The Extended model for the evaluation of coaching [3, 20] differentiates between general proximal outcomes, coaching-specific proximal outcomes, and distal outcomes. General proximal outcome variables are variables used in various areas of personal development and beyond. These variables are, for example, reported satisfaction with coaching and goal attainment [19]. Coaching-specific proximal outcome variables are variables that show the specific characteristics and strengths of coaching compared to other interventions. Such variables used in coaching research are, for example, self-efficacy [21, 22] and self-esteem [23]. Distal outcomes are effects that need time in order to unfold. A distal outcome variable is, for example, life satisfaction [24].

In our research, we chose coaching outcomes described as relevant factors in the coaching meta-analyses [5, 10–15], i.e., satisfaction with coaching, positive and negative affect, self-esteem, self-efficacy, goal attainment, goal-related motivation, and trust. Next to these outcome variables, we chose outcomes described as relevant in general research on counseling and goals, i.e., approach motivation, enhanced understanding, strengthened motivation, and action implementation. In addition, Greif [3, 20] mentions process variables including the coaching relationship, client and coach characteristics, and antecedent variables that make the outcomes of coaching possible.

**Fig 1:**
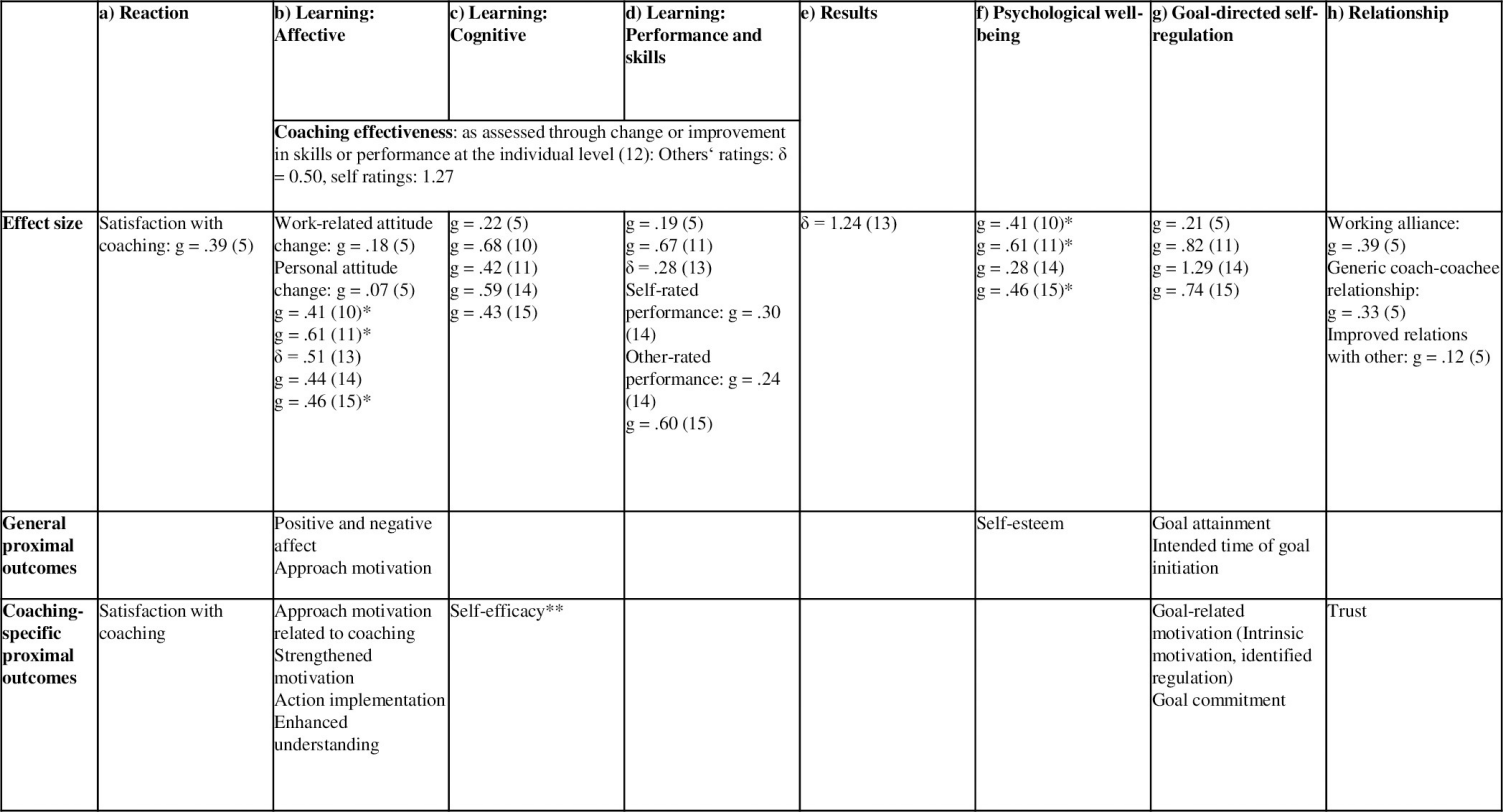
Classification and effect sizes of coaching variables according to the taxonomies used in the coaching meta-analyses and the taxonomy by Greif [3, 20]. The categories are described in the meta-analyses as follows: **Reaction:** *Satisfaction with coaching [5]*. **Learning: Affective**: Work-related attitude change [5]: e.g., motivation, self-efficacy, motivation to transfer coached skills; Personal attitude change [5]: e.g., reduced stress, happiness; *Well-being [10]*: e.g., DASS = Depression, Anxiety and Stress Scale, NAS = Negative Affect Scale, PAS = Positive Affect Scale, SPWB = Scales of Psychological Well-being, SWLS = Satisfaction with Life Scale, WWBI = Workplace Well-being Inventory; *Well-being [11]*: Well-being, hope, resilience, reduced stress, increased life satisfaction, and experienced support; *Affective outcomes [13]:* Attitudes and motivational outcomes (e.g., self-efficacy, well-being, satisfaction); *Affective outcomes [14]*: Attitudinal, commitment and motivational outcomes (e.g., organizational commitment, job satisfaction); *Well-being [15]*: subjective and objective outcome measures that are a direct representation of peoples’ well-being, health, need fulfillment, and affective responses (e.g., measures of psychopathology and burnout). **Learning: Cognitive:** *Cognitive change* [5]: e.g. self-awareness, strategic thinking, emotional intelligence; *Coping [10]*: e.g., Cognitive Hardiness Scale; *preparedness [11]*: e.g. self-awareness, self-efficacy; *Cognitive outcomes [13]:* declarative knowledge; procedural knowledge; cognitive strategies (e.g., problem-solving); *Cognitive outcomes [14]*: knowledge acquisition, knowledge organization and cognitive strategies such as clients’ self-reflection, self-awareness and self-understanding of learning progress and strategy; consisting of a) general perceived efficacy (e.g. self-awareness, self-efficacy) and b) goal attainment; *Coping [15]*: outcome measures related to the ability to deal with present and future job demands and stressors (e.g., self-efficacy, mindfulness). **Learning: Performance and skills**: *Professional skills/performance [11]*: Performance measures (e.g., consultation skills); *Skill-based outcomes [13]:* Compilation and automaticity of new skills (e.g., leadership skills, technical skills, competencies); *Generic behavioral change [5]:* e.g., improved job performance, technical skills, leadership skills, impact and influence; *Skill-based/performance outcomes [14]: D*evelopment of technical skills that links goal; consisting of a) self-rated performance and b) other-rated performance*; Performance/skills [15]*: Subjective and objective outcome measures that either directly reflect performance (e.g. number of sales, supervisory rated job performance) or reflect the demonstration of behaviors needed for an organization to be effective (e.g., transformational leadership behaviors). **Results**: *coaching impact at the organizational level* [12]: e.g., productivity, employee satisfaction; *Results [13]*: Individual, team, and organizational performance (e.g., financial results, objective or goal achievement, productivity). **Psychological well-being:** *Well-being [10]*: e.g., DASS = Depression, Anxiety and Stress Scale, NAS = Negative Affect Scale, PAS = Positive Affect Scale, SPWB = Scales of Psychological Well-being, SWLS = Satisfaction with Life Scale, WWBI = Workplace Well-being Inventory; *Well-being [11]*: Well-being, hope, resilience, reduced stress, increased life satisfaction, and experienced support; *Workplace psychological well-being [14]*: Self-acceptance, purpose in life, positive relations with others, environmental mastery and autonomy (e.g., mental health or resilience); W*ell-being [15]*: subjective and objective outcome measures that are a direct representation of peoples’ well-being, health, need fulfillment, and affective responses” (e.g., measures of psychopathology and burnout). **Goal-directed self-regulation:** *Goal attainment [5]; Goal-effectiveness [11]; Goal attainment [14]:* Trainees’ self-assessment of specific learning outcomes; *Goal-directed self-regulation [15]*: all outcome measures related to goal-setting, goal-attainment, and goal-evaluation. **Relationship:** *Working alliance* [5]; *Generic coach-coachee relationship* [5]: e.g., trust, credibility; *Improved relations with others [5].* *Some variables (e.g. well-being) occur more than once as they can be classified into different categories. **Some authors view it as an affective outcome [5, 13, 17].

In Fig 1, we classified the variables assessed in our studies into the eight categories proposed by the meta-analyses [5, 10–15]: (a) Reaction, (b) Learning: Affective, (c) Learning: Cognitive, (d) Learning: Performance and skills, (e) Results, (f) Psychological Well-being, (g) Goal-directed self-regulation, and (h) Relationship. The effect sizes of the different outcome categories reported in the meta-analyses are also shown in Fig 1. Based on Greif 3, [20], we further differentiated between general proximal outcomes and coaching-specific proximal outcomes.

### Coaching outcome and process variables used in the current studies

Within the category (a) reaction, a typical outcome variable is clients’ satisfaction with coaching. It is usually assessed by asking participants to indicate their subjective satisfaction on a scale [19]. In the current article, three studies report participants’ satisfaction (Studies 2a, 3b, 4a). An affective learning outcome (b) is, for example, positive and negative affect. Within coaching research, it is often assessed using the positive and negative affect schedule [PANAS, 25]. We assess positive and negative affect in Study 3b. Approach motivation also falls into this category. Approach motivation is important for following and attaining a goal set in coaching [26, 27]. It means that people are energized, capable, powerful, and determined to move toward something [28, 29]. Approach motivation can be assessed using self-reports (Studies 1 and 2b within the current article) such as “Right now, I feel energized” [28] but also using implicit measures (Studies 1, 3a, 3c within the current article). A well-established indicator of implicit approach motivation is cerebral asymmetry. Studies using electroencephalogram (EEG) found that relative left frontal activity was associated with self-reported approach motivation [30, for a review]. A behavioral measure to assess frontal activity is the line bisection task [LBT, 31]. Studies using this task [32, 33] take individuals’ biased perception to the right or left visual field when they try to mark the midpoint of horizontal lines. This reflects neural activity in the contralateral hemisphere [34]. Nash and colleagues [34] showed links between the line bisection task and state left frontal activity in EEG. Thus, the line bisection task can be used to assess situational approach-related motivation. According to the Rubicon model of action phases [35], it is important to enhance the clients’ understanding of their actual situation. This helps clients to build a strengthened motivation in order to clarify what they want and to trigger the implementation of an action [36] (Study 4b).

A typical cognitive learning outcome (c) that is often assessed in coaching research is self-efficacy (Studies 3a, 3b, 3c within the current article). It means that individuals believe that are able to master difficult situations [37]. Individuals who believe that they are self-efficient are more likely to exhibit behavior, try harder and persist in their behavior [38]. Coaching research often uses the self-efficacy scale by Jerusalem & Schwarzer [39; e.g., “When I am confronted with a problem, I can usually find several solutions”] which assesses individuals’ general expectation that they are capable of dealing with difficult situations. However, there are also studies that relate self-efficacy to a specific situation or problem [21, 40, 41]. For example, executive coaching increased executives’ self-efficacy expectations related to specific leadership competencies they considered important [41]. In our studies, we did not assess variables that fall into the categories (d) performance and skills [e.g., job performance, 42] or (e) results [e.g., individual productivity, 43] but in Study 3b we assessed one variable which can be categorized into (f) psychological well-being: self-esteem, i.e., an individual’s subjective perceived worth as a person [44]. Within the category (g) “goal-directed self-regulation”, a typical measure in coaching research is the goal attainment scale where clients identify a goal they would like to attain and then rate the degree of goal attainment [10, for an overview]. We assess goal attainment in Studies 1, 3c, and 4b. Another variable that falls within this category is goal-related motivation, including intrinsic motivation and identified regulation (Studies 3a, 3c). According to self-determination theory, motivation can be localized on a continuum of self-integration from extrinsic motivation on the one extreme to identified regulation and intrinsic motivation on the other [45–48]. If individuals identify with a behavior, feelings of autonomy increase. If people have fully integrated a behavior into their self, they act out of intrinsic motivation and feel highly autonomous, which may lead to higher goal acceptance, feelings of owning one’s goals, successful goal attainment, and well-being [48–50]. We also assessed goal commitment (Studies 3a, 3c). It is attained as soon as one has crossed the Rubicon and thus, has made a decision to work on a goal [35]. When individuals are committed to a goal, they make plans how and when they should implement an action [35]. Thus, in one Study (3c), we assessed participants’ intended time of goal initiation.

Within the category “(h) relationship”, trust is an important variable in coaching. Trust in the counselor opens the client to share and explore personal topics [51], an important prerequisite for coaching. Trust can therefore develop and thus, be seen an outcome variable [cf. 5] but it can also be seen as a process variable contributing to a good coach-coachee relationship and thus, to a successful coaching.

### Two self-regulatory systems: Growth versus security

According to regulatory focus theory [RFT, 1], individuals differ in their self-regulatory systems [1, 52]. RFT distinguishes between two independent self-regulatory systems (labeled promotion and prevention) that affect the way individuals perceive the world, what information from the environment they focus on, and which strategies they prefer to pursue their goals. Higgins [1] states that promotion and prevention should not be understood as exclusive categories but rather as independent self-regulatory systems. Therefore, both promotion and prevention can be equally present. Nevertheless, individuals can differ in their expression of the promotion and prevention systems. This is called the dominant regulatory focus [1]. It arises from socialization, that is, from experience, one’s culture, and parenting. For example, for individuals with a dominant promotion focus, parenting may have placed great emphasis on encouraging the child to overcome difficulties or to be confident. The message to the child was that achievements, hopes, and ideals matter in life. For individuals with a dominant prevention focus, parental education may have placed great emphasis on alerting the child to potential dangers or on manners. The message to the child was that what matters in life is safety, a sense of responsibility, and the fulfillment of obligations [1].

Individuals with a dominant *promotion focus* (i.e., promoters) prefer eager advancement strategies when engaging with tasks, and they perceive change processes as opportunities for success. Promoters take risky actions, align their goals and actions to ideals, are particularly sensitive to positive and abstract information from the environment, and are driven by the need for growth. Individuals with a dominant *prevention focus* (i.e., preventers) prefer vigilant, cautious strategies of engaging with tasks and are keen to maintain stability. Preventers perceive possible pitfalls and failures in change processes, align their goals and actions to duties and obligations, are particularly sensitive to negative and concrete information from the environment, and are driven by the need for security [1, 53, 54].

Coaching, as well as counseling, essentially involves a process of personal growth and change [4, 6, 8]. Thus, coaching usually aims at leaving the status quo (0) to approach something new (+1), i.e., goals that the client set and develop within the coaching process. A promotion focus is also associated with a focus on approaching gains (+1), and avoiding the status quo (0), whereas a prevention focus is associated with approaching the status quo (0) and avoiding loss (-1) [54, 55]. Therefore, for individuals with a dominant promotion focus, coaching should be a natural situation, addressing their change-related focus (from 0 to +1). A promotion-related openness to change and a prevention-related preference for stability was shown in several studies. For example, individuals with a dominant promotion focus were more willing to change to a different task instead of continuing an interrupted task and were more willing to exchange an object for another object. Individuals with a dominant prevention focus were more willing to continue an interrupted task and refused to change their received objects [56]. The preventers’ preference for stability over change also fits with the finding that prevention focus is negatively related to personal values representing openness to change [i.e., self-direction and stimulation, 57].

Accordingly, we assume that coaching as a change-oriented process fits the growth-oriented promotion focus more than the security-oriented prevention focus. This should become apparent in a better evaluation of coaches and coachings in general and a higher coaching success.

H1: Clients with a higher promotion than prevention focus evaluate coaching better and report more coaching success than clients with a higher prevention than promotion focus.

### Regulatory fit

Not only focus alone influences goal pursuit but also the fit between regulatory focus and strategies or situational means to reach a goal. This fit is called regulatory fit [2]. For example, a series of studies matched arguments concerning sociopolitical or health care issues to an individual’s regulatory focus [58–60]. They found that regulatory fit leads arguments to be perceived as more persuasive and increases people’s intention to act in line with the arguments’ recommendations. That is, after receiving growth- or gain-oriented arguments, promotion-focused individuals were more likely to initiate behavioral change than after receiving security- or loss-oriented arguments, whereas the opposite pattern was found for prevention-focused individuals. Moreover, when an incentive in an anagram task was framed in terms of gaining money (promotion framing) rather than in terms of losing money (prevention framing), promotion-oriented students performed better. For prevention-oriented students, the reverse was found [52]. Regulatory fit can also positively influence an individual’s motivation following feedback: Promoters showed higher motivation after positive feedback about previous successful work steps whereas preventers showed higher motivation when the feedback was based on negative indicators, such as previous errors or non-achieved targets [61]. Additionally, preventers showed more adaptive behavior and commitment towards a change when it was communicated by concentrating on the avoidance of possible mistakes rather than by concentrating on possible ways in which the change could promote professional growth [62]. A meta-analysis has shown medium effects of regulatory fit on evaluation, behavioral intention, and behavior [63]. While promotion fit compared to prevention fit showed a stronger effect on evaluation, prevention fit compared to promotion fit showed a stronger effect on behavior. Promotion focusing more on rewards may increase the value of a product and thus, the evaluation. Prevention focusing more on failures and mistakes may increase their focus on the outcomes, and thus, on the behavior [63].

Although RFT has been researched intensively, studies testing it in the context of coaching or counseling are scarce. One study indicated that a fit between a coach’s and a client’s regulatory focus may affect an individual’s performance in line with the regulatory fit hypothesis [64]. While clients generally benefited from a coach who highlighted previous partial successes and further opportunities for improvement, clients who perceived their personality traits as rather unchangeable (which indicates a prevention focus) benefitted most from a coach who highlighted previous mistakes and problems [a prevention-focused strategy; 64]. Accordingly, we suggest that if coaching is experienced as congruent with the client’s chronic regulatory focus, there will be also a positive impact on factors that are relevant to coaching. In this regard, even a preventer’s commitment towards change can be increased when the situation is congruent with his or her regulatory focus [cf 62]. In line with this, Taylor-Bianco and Schermerhorn [65] present a model in the context of leadership in which motivation to pursue change goals is predicted by regulatory fit. Although, in general a promotion focus relative to a prevention focus is related to a higher motivation to change, a prevention focus may lead to motivation to change when the change-situation requires stability. In their model they claim that prevention-oriented leaders are more likely to pursue change goals when they are in an environment of stability compared to an environment of rapid and unpredictable change.

Therefore, we hypothesize that coachings emphasizing prevention-related aspects, e.g., avoiding mistakes and risks to achieve stability and security, are more beneficial for clients with a higher prevention than promotion focus. Coachings emphasizing promotion-related aspects, i.e., exploiting opportunities and challenges to achieve growth, are more beneficial for clients with a higher promotion than prevention focus:

H2: A fit between the regulatory focus of the client and the regulatory focus of the coaching positively affects coaching success. That is, promotion-focused coaching approaches or coaching interventions are more beneficial for promotion-focused clients and prevention-focused coaching approaches or coaching interventions are more beneficial for prevention-focused clients.

Given the existing literature on the positive effects of regulatory fit in different areas, the effectiveness of a regulatory fit should exist also in coaching. However, there is little research on this topic and we are unclear about the importance of regulatory fit for coaching and about which coaching variables are particularly affected. A meta-analysis of regulatory fit including 215 studies with a total of 23,690 participants shows small to medium effects of regulatory fit on evaluation, behavioral intention, and behavior [63] and meta-analyses on coaching report small to large effect sizes of coaching on various outcomes (Fig 1). Thus, the aims of the current studies are to show the effect of regulatory fit in coaching and to figure out which outcome variables are particularly affected.

### Interpersonal regulatory fit

Apart from regulatory fit developing when individual’s focus aligns with a situation or task, there is also the concept of interpersonal fit. Interpersonal fit is experienced when the regulatory foci of two individuals match. Righetti et al. [66] examined interpersonal regulatory fit during goal pursuit activities and found in six studies that promotion-oriented individuals benefitted from interpersonal fit. Thus, when they interacted with a promotion-oriented compared to a prevention-oriented partner they reported more enjoyment, a higher feeling right about their goal pursuit, and a higher motivation regarding goal pursuit. Furthermore, promotion-oriented individuals evaluated a promotion-oriented partner as more instrumental, useful, and helpful to achieve their goals than a prevention-oriented partner. Prevention-oriented individuals did not benefit from such interpersonal fit [66]. As coaching is a social interaction in which coach and coachee work together to achieve goals, we hypothesize that when there is an interpersonal regulatory fit between coach and coachee, coaching success is higher.

H3: A fit between the client’s and coach’s regulatory focus positively affects coaching success. This means that clients with a dominant promotion focus benefit most from coaches with a dominant promotion focus and clients with a dominant prevention focus benefit most from coaches with a dominant prevention focus.

### Value from fit within coaching

Regulatory fit theory [2] proposes that individuals experience a feeling right when the manner in which they pursue a goal fits their regulatory focus. The feeling-right-experience translates to an increased perceived value of what people are currently doing which is called value from fit [67–69]. As a result, self-regulatory processes are facilitated, the relevance of the information processed is increased and self-integration of the information may proceed [69, 70]. As research shows [2, 69–73], value from fit manifests in an increased *inclination* toward a behavior, an increased *motivation* to engage in a behavior, *positive prospective feelings* about a behavior, *positive retrospective feelings* about a behavior and an increased *value* of a behavior. This experience has been shown to mediate positive effects of regulatory fit on behavior [58]. Similarly, it can be assumed that a coaching that is in line with the client’s chronic regulatory focus feels right and thus, leads to an increased value of the coaching activity. This should in turn increase coaching success. Therefore, we hypothesize:

H4: The client’s value from fit mediates the effect of regulatory fit on coaching success.

### Overview of the studies

As literature defines coaching as a change- and growth-oriented process [4, 6, 8], we first examine our hypothesis that coaching fits the growth-oriented promotion focus more than the security-oriented prevention focus. This should mean that clients with a higher promotion than prevention focus evaluate coaching better and report more coaching success than clients with a higher prevention than promotion focus (H1, Studies 1, 2, 4a).

Given the existing literature on persuasion, feeling right, fluency, and processing styles as a function of regulatory fit, the effectiveness of a regulatory fit should exist also in coaching (H2). Thus, we tested whether matching the coaching approach (Study 2) or coaching intervention (Studies 3a, 3b, 3c) with the client’s regulatory focus results in higher goal attainment and commitment, more goal-related motivation, more approach motivation, more self-efficacy, higher self-esteem, and more positive affect. As research has also shown motivational benefits of interpersonal fit regarding goal pursuit [66], we tested whether the fit between the coach’s and the client’s regulatory focus also creates benefits in coaching (H3, tested in Studies 4a and 4b). Moreover, we examined whether the mediating effect of individuals’ value from fit as already shown in regulatory fit research [67–69] can be replicated for coaching (H4, tested in Studies 2 and 3c).

Thus, the current line of studies has two aims. First, to show the benefits of regulatory fit also in the area of coaching and second, to show which coaching variables are particularly affected. All studies were approved by the University of Salzburg ethical committee.

#### Transparency, openness, and statistical analyses

For all studies, we describe our sampling plans, all data exclusions, all manipulations, and all measures (The measures for all studies are described in detail in S1 Table). All data and analysis codes are available at https://osf.io/f7uec/?view_only=0e25fdcbab94468aae9df807e0967f33. Materials for the studies are available by emailing the corresponding author. The studies’ designs and their analyses were not preregistered. All statistical analyses were performed using SPSS (version 27, IBM, Armonk, NY). Results are reported as significant when they reach a significance level of 5% and reported as “failed to reach the significance level of 5%” when they reach a significance level of 10%. For calculating Olkin’s z, we used an online calculator [74].

For all studies except of Study 4b, we used questionnaires assessing regulatory focus. Although prevention focus and promotion focus are theoretically independent, it has become common and accepted practice in previous studies to distinguish between promoters and preventers based on their regulatory focus index (RFI), which is a difference score of the two scales [promotion minus prevention score, 75, 76]. Positive values indicate a relatively higher promotion than prevention focus. Individuals with positive scores are therefore called “promoters” or “individuals high on promotion” and individuals with negative scores “preventers” or “individuals high on prevention.” Although some propose that promotion and prevention are two distinct strategies that coexist [77], others also create a difference score which gives us the opportunity of clearly distinguishing promotion-oriented from prevention-oriented individuals [62]. In our studies, we report both, the separate scales as well as the difference score. Although we are aware of the statistical and methodical implications of working with difference scores [78], for the ease of interpretation of the results, especially for the moderation analyses in Studies 3a, 3b, 3c and 4b, we use the difference score.

## Study 1

In this study, we investigate the hypothesis that coaching clients with a higher promotion than prevention focus report more coaching success than coaching clients with a higher prevention than promotion focus (H1). We thereby examined data from a longitudinal coaching study consisting of five coaching sessions per client and explicit and implicit measures of coaching success.

### Method

#### Participants

The study was part of a psychology course for master students. In this course, master psychology students received 220 hours of career coaching training according to a resource- and solution-focused career coaching concept [79]. Thus, coaches were master students who offered a career coaching with five sessions. Two hundred and seventy-one of their coaching clients filled out an online questionnaire before they participated in the coaching. As data was collected both online as well as in paper-pencil form, our sample varies according to analysis. Of the 271 coaching clients (*M*_age_ = 25.25 years, *SD* = 5.90; 172 female, 78 male, 21 did not indicate gender) who had completed the online regulatory focus questionnaire before the start of the first coaching session, 103 clients indicated both, their target and final goal attainment (114 indicated target goal attainment, 108 indicated final goal attainment). Of the 271 clients, 107 right-handed clients completed the implicit approach motivation before session 1 and after session 5. Of the 271 clients, 96 completed both, the baseline and final self-reported approach motivation (107 indicated baseline approach motivation and 120 indicated final approach motivation).

Since we did not perform an a-priori power analysis for this study, we ran a sensitivity analysis using G*Power [80]. With a minimum sample size of *N* = 103, we were able to detect an effect size of *r* = .24 with a power of .80, *r* = .26 with a power of .85, *r* = .28 with a power of .90, and *r* = .32 with a power of .95, indicating that the study had sufficient power to detect the found correlations between RFI and final goal attainment (*r* = .30) and RFI and implicit approach motivation (*r* = .26), but was underpowered to detect the found correlation between RFI and goal discrepancy (*r* = .19).

#### Procedure and measures

The coaching clients participated in five bi-weekly career coaching sessions each lasting about 1.5 hours. One week before the first coaching session, clients were sent a link to an online questionnaire assessing their regulatory focus. At the beginning of the coaching, clients provided written informed consent. Immediately before the first coaching session, we assessed clients’ implicit and self-reported approach motivation either using a paper-pencil or an online questionnaire. For clients’ implicit approach motivation, we used the line bisection task [LBT, 31]. The task involved 10 staggered horizontal lines of different length on a sheet of paper or on the computer. Participants were instructed to mark the middle of each line with a pen or the cursor. We calculated the distance of participants’ marks from the objective midpoint. Rightward errors were scored as positive values and leftward errors were scored as negative values. By averaging the scores across the 10 lines, we built the mean LBT scores with positive scores indicating relatively more rightward errors and thus approach motivation. As we were interested in people’s change in their motivational direction of behavior, we built a difference score reflecting the change from the beginning of the coaching to the end of the coaching (final-baseline approach motivation). Positive values indicate an increase in approach motivation. In line with prior research using the LBT and to control for hemispheric differences arising from handedness [81, 82], we only selected right-handed participants. During the first coaching session when clients had written down their goals, the coach asked them to indicate their target goal attainment on a scale from 1 to 10 and noted this value. At the end of the fifth session, the coach asked clients to indicate their actual goal attainment and gave them a paper-pencil questionnaire or an online questionnaire assessing their implicit and self-reported approach motivation. Clients immediately answered this questionnaire. All measures are described in detail in S1 Table. Apart from the measures used to test our main hypothesis, we included supplementary measures for additional research questions. As those measures are not part of the current research question, they are available from the authors upon request.

### Results and discussion

For means, standard deviations, and correlations see Table 1.

**Table 1:** Means, standard deviations, and correlations for Study 1.

Variable	*M*	*SD*	1	2	3	4	5	6	7	8	9	10	11	12	13	14
1. RFI (Promotion-Prevention)	0.03	0.78	-													
2. Promotion	3.73	0.49	.73**	-												
3. Prevention	3.70	0.54	-.79**	-.16(*)	-											
4. Final goal attainment (after session 5)	7.05	1.64	.30**	.27**	-.20*	-										
5. Target goal attainment (session 1)	8.09	1.54	.09	.07	-.06	.45**	-									
6. Goal discrepancy (final—target goal attainment)	-1.09	1.67	.19(*)	.21*	-.09	.09	-.48**	-								
7. Implicit approach motivation before session 1 (baseline)	-0.43	1.55	-.10	-.08	.07	.16	.17	-.01	-							
8. Implicit approach motivation after session 5 (final)	-0.18	1.67	.20*	0.22*	-.10	.21	.07	.14	.32**	-						
9. Implicit approach motivation difference (final-baseline)	0.24	1.87	.26**	.26**	-.15	.06	-.08	.14	.54**	.63	-					
10. Self-reported approach motivation before session 1 (baseline)	3.45	0.65	.20*	.33**	.01	.19	-.04	.27	.02	.11	.09	-				
11. Self-reported approach motivation "determined" before session 1 (baseline)	3.66	0.97	.19(*)	.27**	-.03	.19	-.01	.22	.07	.11	.05	.65**	-			
12. Self-reported approach motivation after session 5 (final)	4.04	0.76	.18(*)	.27**	.003	.15	-.09	.24(*)	-.11	.07	.15	.50**	.48**	-		
13. Self-reported approach motivation "determined" after session 5 (final)	4.19	0.79	.07	.15	.05	.08	-.16	.21	-.15	-.04	.08	.44**	.42**	.76**	-	
14. Self-reported approach motivation difference (final-baseline)	0.57	0.81	.04	-.002	-.06	-.02	-.11	.11	-.16	-.001	.12	-.43**	-.05	.66**	.42**	-
15. Self-reported approach motivation "determined" difference (final-baseline)	0.52	1.01	-.09	-.13	.02	-.15	-.14	-.03	-.23*	-.14	.05	-.24*	-.66**	.20	.44**	.39**

(*) *p* < .10

* *p* < .05

** *p* < .01

#### Descriptives

At the end of the coaching, individuals attained a mean goal attainment value of *M* = 7.05. There was still a mean discrepancy to their target goal attainment of *M* = 8.09, indicating that in total, clients did not fully attain their goals. As clients’ implicit approach motivation was more negative at the beginning of the coaching (baseline: *M* = -0.43) than at the end (final: *M* = -0.18), and their self-reported approach motivation was lower at the beginning (baseline: *M* = 3.45/3.66) than at the end of the coaching (baseline: *M* = 4.04/4.19), the results suggest that there was an increase in approach motivation.

#### Correlations

Regarding *goal attainment*, the correlations between RFI and the *target goal attainment score* were non-significant, indicating that promoters did not set themselves higher goals than preventers (see Table 1, for the exact values). We found a significant positive correlation between RFI and *final goal attainment*, *r*(106) = .30, *p* = .001, indicating that a higher promotion than prevention focus was associated with higher goal attainment at the end of the coaching. Olkin’s z for the comparison between the correlations of promotion and prevention with goal attainment (promotion: *r*(106) = .27, *p* = .004; prevention: *r*(106) = -.20, *p* = .042) shows a significant difference, *z* = 3.44, *p* < .001, hinting at a promotion focus being associated with higher goal attainment than a prevention focus. The correlation of RFI with the *goal discrepancy* was at the border of the significance level of 5%, *r*(101) = .19, *p* = .050. Olkin’s z for the comparison between the correlations of promotion and prevention with the goal attainment score (promotion: *r*(101) = .21, *p* = .031; prevention: *r*(101) = -.09, *p* = .354) shows a significant difference, *z* = 2.06, *p* = .020, hinting at a promotion focus being associated with lower goal discrepancy than a prevention focus.

Regarding *implicit approach motivation*, we first checked whether a higher promotion than prevention focus was associated with more approach motivation already at the beginning of the coaching. The correlations of RFI with the ba*seline score of implicit approach motivation* were non-significant, indicating that in the beginning of the coaching, promoters had not been more approach motivated than preventers (see Table 1, for the exact values). The correlations of RFI with *final approach motivation*, *r*(105) = .20, *p* = .037 and with the *difference score (final-baseline approach motivation)* were significant, *r*(105) = .26, *p* = .006, indicating that a higher promotion than prevention focus was associated with more implicit approach motivation at the end of the coaching and with more increase in approach motivation. Olkin’s z for the comparison between the correlations of promotion and prevention with the final approach motivation showed a significant difference (promotion: *r*(105) = .22, *p* = .026; prevention: *r*(105) = -.10, *p* = .316), *z* = 2.25, *p* = .012, and also the correlations of promotion and prevention with the difference score showed a significant difference (promotion: *r*(105) = .26, *p* = .007; prevention: *r*(105) = -.15, *p* = .133), *z* = 2.94, *p* = .002. Thus, a promotion focus seems to be associated with more approach motivation than a prevention focus.

Regarding *self-reported approach motivation*, we first checked whether a higher promotion than prevention focus was associated with more self-reported approach motivation already at the beginning of the coaching. The correlations of RFI with the baseline score of self-reported approach motivation were significant (self-reported approach motivation before session 1 (baseline): *r*(105) = .20, *p* = .041; self-reported approach motivation "determined" before session 1 (baseline): *r*(105) = .19, *p* = .050), indicating that in the beginning of the coaching, promoters reported more approach motivation than preventers.

Regarding the *final self-reported approach motivation*, the positive correlation between RFI and final approach motivation failed to reach the significance level of 5%, *r*(118) = .18, *p* = .056. Olkin’s z for the comparison between the correlations of promotion and prevention with the final approach motivation showed a significant difference (promotion: *r*(118) = .27, *p* = .003; prevention: *r*(118) = .003, *p* = .977), *z* = 1.99, *p* = .023, indicating that a promotion was associated with more self-reported approach motivation at the end of the coaching than prevention. The correlation between RFI and the single item “determined” at the end of session 5 was not significant, *r*(118) = .07, *p* = .482. The correlations of RFI with the difference scores were not significant (self-reported approach motivation difference and self-reported approach motivation "determined" difference, see Table 1).

Summarized, there were significant positive correlations of RFI with final goal attainment and the goal discrepancy score, and final implicit approach motivation and the increase in implicit approach motivation from the start to the end of the coaching. Thus, regarding these variables, individuals scoring high on promotion may benefit more from coaching than individuals high on prevention. However, for final and the increase in self-reported approach motivation, the correlations did not reach significance. Here, the baseline scores were significantly correlated with RFI, indicating that promoters felt more energized, powerful, capable, goal-focused, and determined than preventers right away. This is in contrast to the non-significant correlation of RFI with baseline implicit approach motivation. The two approach motivation measures may thus reflect distinct constructs, especially since they do not correlate with each other (see Table 1). We address this issue in the general discussion.

The findings partly support H1 such that regarding goal attainment and having an implicit “impulse to move toward” [30, p 292], people scoring high on promotion benefit more from coaching than people high on prevention. For self-reported approach motivation, the results did not show significance.

#### Correction for multiple comparisons

As for our hypothesis H1, we test four dependent variables, i.e., goal attainment, implicit approach motivation, self-reported approach motivation, self-reported approach motivation “determined”, we performed an alpha correction for multiple comparisons. Controlling for the family-wise error rate, we applied the Bonferroni correction with an alpha level of .05 and four tests. This results in a corrected alpha level of *p* = .013. Interpreting the findings with the corrected alpha level, only the correlation of RFI with the implicit approach motivation difference and final goal attainment remains.

## Study 2

As Study 1 partly shows that coaching fits promoters more than preventers, we further test this hypothesis in Study 2. We examine whether individuals are in general more interested in coaching than prevention-oriented individuals, resulting in a better evaluation of coachings (H1). Next, we investigate the hypothesis that a fit between the client’s regulatory focus and the regulatory focus of the coaching positively affects the individuals’ evaluation of coachings (H2). In Study 2, participants read the descriptions of three coachings–a promotion-oriented, a prevention-oriented, or a neutral coaching–and evaluated them by indicating their satisfaction with the coachings, their approach motivation, and their value from fit regarding the coachings. Research suggests that individuals derive value from regulatory fit, a corollary of increased hedonic experience and thus, feeling right about what one is currently engaged in [58, 67–69]. Further, value derived from regulatory fit may result in increased relevance and amplified value regarding the information processed [69, 70, 83]. Thus, we investigated whether people’s experience of value from fit mediate the effect of regulatory focus on people’s satisfaction with the coaching offers (H4).

### Method

#### Participants

To determine the minimum sample size required to test our fit-hypothesis (H2), we conducted an a priori power analysis using G*Power [80]. Results indicated that for detecting a small to medium effect (*r* = .25) and achieving a power of 80% at α = .05, the required sample size was *N* = 97. We obtained a sample size of 99 German-speaking participants who were interested in coaching (41 females and 53 males, 5 did not indicate their gender). They were recruited for an online study via personal contacts. In terms of profession, the sample was very heterogeneous with participants working in sectors such as production, health, information and communication, or service providers. Participants’ age ranged from 18 to 70 years with *M*_age_ = 37.15 years (*SD* = 14.41).

#### Procedure and measures

After filling out a measure for the regulatory focus trait questionnaire, participants were instructed to imagine being offered coaching for personnel development. Then, participants could choose one out of five topics matching their actual professional situation. The topics were management responsibility, a new position, self-reflection, leadership, and personal development. They were shortly described. Based on their topic choice, they were presented with the description of three coachings—a promotion-oriented coaching, a prevention-oriented coaching, and a neutral coaching. The different coachings were presented in randomized order. Please see S1 File for a coaching example. Then, participants were asked to indicate their self-reported approach motivation and their satisfaction regarding the three coachings. As a mediator variable, we assessed individuals’ value from fit. Conceptually following Latimer et al. [58] and adapting their approach to the coaching context, we constructed 5 items, with each item measuring one of the aspects: *inclination* towards participating in the coaching (i.e., “Participating in this coaching will give me joy”), *motivation* to participate in the coaching (i.e., “I’m motivated to participate in this coaching”), *positive prospective feelings* about participation in the coaching (i.e., “It will feel good to participate in this coaching”), as well as *positive retrospective feelings* about participation in the coaching (i.e., “Once I have participated in the coaching, I will feel positive about it”) and *perceived value* of the participation in the coaching (i.e., “The participation in the coaching is valuable to me”). In addition, we integrated the item “It feels right to participate in this coaching” into the scale [e.g., 68]. Finally, they answered demographic questions. All measures are described in detail in S1 Table.

### Results

For means, standard deviations, and correlations see Table 2.

**Table 2:** Means, standard deviations, and correlations for Study 2.

Variable	*M*	*SD*	1	2	3	4	5	6	7	8	9	10	11	12	13	14	15
1. RFI (Promotion-Prevention)	0.14	1.25	-														
2. Promotion	5.32	0.84	.70**	-													
3. Prevention	5.18	0.89	-.74**	-.04	-												
4. Satisfaction (all coachings)	5.37	1.15	.21*	.30**	-.01	-											
5. Satisfaction (Promotion coaching)	5.56	1.43	0.20*	.27**	-.03	.66**	-										
6. Satisfaction (Prevention coaching)	5.14	1.60	0.14	.27**	.06	.77**	.21*	-									
7. Satisfaction (Neutral coaching)	5.40	1.57	0.14	.14	-.06	.81**	.32**	.48**	-								
8. Value from fit (all coachings)	5.15	1.20	.19(*)	.31**	.03	.91**	.57**	.71**	.76**	-							
9. Value from fit (Promotion coaching)	5.39	1.32	.25*	.30**	-.07	.63**	.87**	.23*	.35**	.70**	-						
10. Value from fit (Prevention coaching)	4.82	1.68	.07	.27**	.15	.74**	.22*	.90**	.49**	.81**	.31**	-					
11. Value from fit (Neutral coaching)	5.23	1.59	.14	.16	-.04	.78**	.34**	.47**	.91**	.84**	.43**	.52**	-				
12. Approach motivation—"determined" (all coachings)	5.27	1.21	.19(*)	.35**	.07(*)	.88**	.62**	.68**	.69**	.86**	.61**	.71**	.68**	-			
13. Approach motivation—"determined" (Promotion coaching)	5.43	1.57	.26**	.32**	-.07	.47**	.82**	.10	.18(*)	.46**	.82**	.15	.20(*)	.63**	-		
14. Approach motivation—"determined" (Prevention coaching)	4.98	1.83	.05	.22*	.13	.70**	.23*	.88**	.42**	.70****	.27**	.91**	.41**	.77**	.20(*)	-	
15. Approach motivation—"determined" (Neutral coaching)	5.39	1.65	.10	.21*	.06	.71**	.31**	.40**	.87**	.67**	.27**	.43**	.85**	.74**	.22*	.38**	-

(*) *p* < .10

* *p* < .05

** *p* < .01

To test H1, hypothesizing that a higher promotion than prevention focus is associated with a better evaluation of coachings, we averaged the responses for the scales approach motivation and satisfaction over the promotion, the prevention, and the neutral coachings. There was a significant positive correlation between RFI and satisfaction, *r*(99) = .21, *p* = .037, indicating that a higher promotion than prevention focus was associated with more satisfaction with coaching. The correlation between RFI and approach motivation did not reach the significance level of 5%.

For the fit-hypothesis H2, RFI shows significant positive correlations with people’s self-reported approach motivation, *r*(97) = .26, *p* = .009, their satisfaction with the promotion coachings, *r*(97) = .20, *p* = .049, and their value from fit regarding the promotion coachings, *r*(97) = .25, *p* = .013, i.e. higher scores on RFI (i.e., higher promotion than prevention focus) are associated with more approach motivation, more satisfaction, and more value from fit regarding the promotion coachings and vice versa for lower scores on RFI. The correlations of RFI with the prevention and neutral coachings are not significant. Olkin’s z for the comparison between the correlations of RFI with the promotion coachings and RFI with the prevention coachings show a significant difference in approach motivation, *z* = 1.70, *p* = .044, indicating that a heightened promotion focus seems to be associated with more and a heightened prevention focus with less approach motivation. However, the comparisons failed to reach significance on value from fit, *z* = 1.57, *p* = .05961, and satisfaction, *z* = 0.49, *p* = .314.

Looking at the correlations of the separate promotion and prevention scores with the dependent variables, a high promotion focus is positively correlated with satisfaction, approach motivation, and feeling right regarding the promotion coaching as well as the prevention coachings and even with approach motivation for the neutral coaching. Thus, individuals scoring high on promotion seem to be satisfied with all kinds of coaching. There were no significant correlations between prevention and the dependent variables.

#### Correction for multiple comparisons

As for our hypothesis H2, we test three dependent variables, i.e., satisfaction, value from fit, and self-reported approach motivation, we performed an alpha correction for multiple comparisons. Controlling for the family-wise error rate, we applied the Bonferroni correction with an alpha level of .05 and three tests. This results in a corrected alpha level of *p* = .017. Interpreting the findings with the corrected alpha level, the significant correlations of RFI with people’s approach motivation and their value from fit regarding the promotion coaching remain but the correlation of RFI with satisfaction with the promotion coaching disappears.

#### Mediation

Investigating H4 hypothesizing that value from fit regarding the coaching mediates the fit between the client’s regulatory focus and the regulatory orientation of the coaching on coaching success, we performed a mediation analysis using the software PROCESS 3.4.1 [84, model 4]. As there were significant correlations of RFI with promotion coachings but not with prevention or neutral coachings, we performed the mediation only for the promotion coachings. The criterion for detecting a mediation was a significant indirect effect which was computed using a 95% bias-corrected bootstrap confidence interval (95% BC CI) and 10,000 bootstrap samples. Regression analyses revealed that RFI had significant total effects on satisfaction with the promotion coachings, *b* = .23, *SE* = .11, *t*(97) = 1.99, *p* = .049, and on self-reported approach motivation regarding the promotion coachings, *b* = .33, *SE* = .13, *t*(97) = 2.67, *p* = .009. The effects decreased to non-significance when the mediator value from fit had been added to the predictions, *b* = -0.02, *SE* = .06, *t*(97) = -0.41, *p* = .686 (for satisfaction), *b* = 0.08, *SE* = .08, *t*(97) = 1.01, *p* = .314 (for approach motivation). RFI positively affected the mediator value from fit, *b* = 0.26, *SE* = .10, *t*(97) = 2.54, *p* = .013. The mediator value from fit positively affected satisfaction with the promotion coachings, *b* = 0.96, *SE* = .07, *t*(97) = 13.36, *p* < .001, and approach motivation regarding the promotion coachings, *b* = 0.96, *SE* = .06, *t*(97) = 17.00, *p* < .001. The bootstrapped indirect effects of RFI via value from fit was significant for satisfaction with the promotion coachings, *b* = 0.25, *SE* = .12, BC CI [0.01, 0.48] and for approach motivation regarding the promotion coachings, *b* = 0.25, *SE* = .12, BC CI [0.01, 0.47]. In sum, the perception of value from fit fully mediated satisfaction and approach motivation regarding the promotion coachings for individuals with a more pronounced promotion focus.

### Discussion

Summarized, in Study 2 we found that promotion-focused clients are more satisfied with coaching in general than prevention-focused clients, providing support for H1. Considering regulatory fit, a higher promotion than prevention focus (RFI) is associated with a higher satisfaction with the promotion coaching and in addition with heightened approach motivation and a value from fit regarding promotion coaching and vice versa for a higher prevention than promotion focus. This result only partially provides support for H2 as we did not find significant correlations of RFI with the prevention coachings. Value from fit mediated the promoters’ satisfaction and approach motivation regarding the promotion coaching. As we did not find a mediation for individuals with a more pronounced prevention focus, the result only partly supports H4.

Study 2 shows small effects only for promoters. While there were correlations of a more pronounced promotion than prevention focus with satisfaction and “determination” regarding a promotion coaching, there were no correlations with prevention coachings or neutral coachings. The promotion fit effects were mediated by promoters’ increased value from fit regarding promotion coachings. However, the vignette-based approach is certainly a critical issue that we discuss in the general discussion section.

In summary, looking at the results of Study 2, one can cautiously say that for offering coachings, the regulatory focus should be considered because fit effects can better explain people’s attitudes and motivation regarding a coaching than a general evaluation of coachings. This is in line with a study revealing fit effects in the context of job offers. Here, promoters were more attracted by job offers containing the promotion-related value autonomy at work and preventers were more attracted by job offers containing the prevention-related value security at work [85]. We find similar fit effects in our study but only for promoters. But is there also a way to establish a regulatory fit in coaching? We investigate this question in the next three studies by exploring the effect of promotion- or prevention-versions of established coaching interventions (Studies 3a, 3b, 3c).

## Study 3a

In this study, we focused on the action planning phase of a coaching process which typically takes place at an early stage in the coaching process. We created an intervention using growth- or security-oriented goal-setting strategies. We hypothesized that promotion-focused coaching clients benefit more from identifying promotion-oriented action strategies to reach their goal and prevention-focused coaching clients benefit more from identifying prevention-oriented action strategies to reach their goal.

### Method

#### Participants

To determine the minimum sample size required to test our fit-hypothesis (H2), we conducted an a priori power analysis using G*Power [80]. Results indicated that for detecting a small to medium effect (f^2^ = .10) and achieving a power of 80% at α = .05, the required sample size was *N* = 81. We obtained a sample size of 120 German-speaking participants (80 females and 40 males) who were recruited for an online study via university newsletters, social networks, or personal contacts. The survey language was German. Participants’ age ranged from 16 to 64 years with *M*_age_ = 25.78 years (*SD* = 7.52). Students in the sample received course credit in exchange for their participation. Participants were randomly assigned to either the promotion (*n* = 56) or prevention coaching intervention (*n* = 64).

#### Procedure and measures

At the beginning of the study, participants answered the regulatory focus questionnaire and were asked to write down a personal goal they would like to attain in the next 4 weeks. This was followed by an explanation of the SMART method of goal setting, which is a coaching technique used to properly define and operationalize goals [86]. It was at this point that the actual manipulation took place. In the promotion coaching intervention, participants were asked to identify promotion-oriented action strategies to reach their goal, whereas in the prevention coaching intervention participants were asked to identify prevention-oriented action strategies to reach their goal. The instructions were as follows:

Promotion coaching intervention: *“Now try to describe the goal you defined before in accordance with the SMART criteria*. *Then think about which actions are necessary to reach your goal*. *Please list at least three actions and describe them briefly*.*”*

Prevention coaching intervention: *“Now try to describe the goal you defined before in accordance with the SMART criteria*. *Then think about which actions you should definitely avoid reaching your goal*. *Please list at least three actions and describe them briefly*.*”*

After working on the coaching intervention, participants completed the LBT to assess approach motivation. They filled out a questionnaire assessing self-efficacy, goal-related motivation, and goal commitment before finishing the survey with another LBT and a set of demographic questions. All measures are described in detail in S1 Table.

#### Statistical analysis

**Fig 2:**
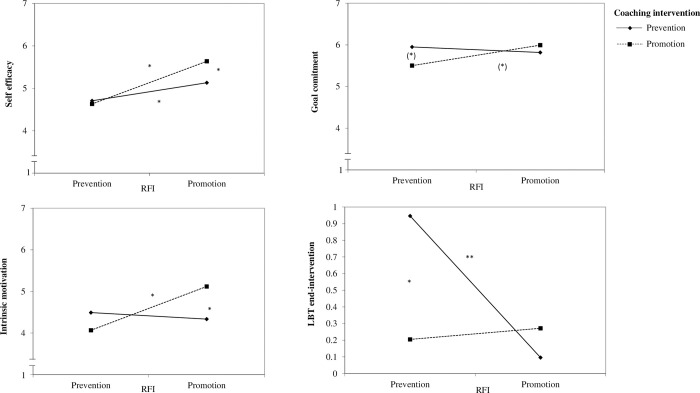
Interaction effects of RFI and coaching intervention on the dependent variables in Study 3a. (*) *p* < .10; * *p* < .05; ** *p* < .01.

To test H2b we used moderated regression analyses of the SPSS macro PROCESS with a 95% bias-corrected bootstrap confidence interval (95% BCCI) and 10,000 bootstrap samples [84, model 1]. We used the coaching intervention condition (coded with prevention = 0, promotion = 1) as an independent variable and people’s RFI as a moderator variable. For significant results, follow-up simple slope analyses are investigated using the conditional effect of the intervention condition on the dependent variables for promoters (+1 SD of RFI, i.e., dominant promotion focus) and preventers (–1 SD of RFI, i.e., dominant prevention focus). See Table 3 for the estimates and Fig 2 for the interaction effects.

**Table 3:** Estimates for RFI, the intervention and their interaction for Study 3a.

Variable	R^2^	Predictor	*b*	Lower 95% CI	Upper 95% CI	*SE*	*t*	*p*
Self-efficacy	.20		* *			* *		
		**RFI**	**0.19**	**0.03**	**0.35**	**0.08**	**2.30**	**.023**
		Intervention	0.23	-0.07	0.52	0.15	1.53	.129
	R^2^ change: .02	**RFI x Intervention**	**0.26**	**-0.02**	**0.53**	**0.14**	**1.86**	**.066**
Intrinsic motivation	.05							
		RFI	-0.07	-0.38	0.25	0.16	-0.41	.680
		Intervention	0.21	-0.36	0.77	0.29	0.72	.475
	R^2^ change: .03	**RFI x Intervention**	**0.54**	**0.01**	**1.07**	**0.27**	**2.03**	**.044**
Identified regulation	.01							
		RFI	0.06	-0.15	0.26	0.11	0.53	.598
		Intervention	0.04	-0.41	0.33	0.19	-0.23	.823
	R^2^ change: .001	RFI x Intervention	0.07	-0.28	0.42	0.18	0.41	.685
Goal commitment	.03							
		RFI	-0.06	-0.25	0.13	0.10	-0.65	.518
		Intervention	0.12	-0.46	0.22	0.17	-0.69	.495
	R^2^ change: .025	**RFI x Intervention**	**0.28**	**-0.04**	**0.60**	**0.16**	**1.74**	**.085**
Implicit approach motivation	.08							
		**RFI**	**-0.38**	**-0.65**	**-0.11**	**0.14**	**-2.75**	**.007**
		Intervention	-0.26	-0.74	0.22	0.24	-1.08	.282
	R^2^ change: .03	**RFI x Intervention**	**0.41**	**-0.05**	**0.86**	**0.23**	**1.78**	**.078**

Significant effects at *p* < 0.05 and effects that reached a significance level of *p* < 0.10 are in bold. Coding of coaching intervention: 0 = prevention, 1 = promotion.

### Results

#### Self-efficacy

The results revealed a significant main effect of RFI, indicating that overall people high on promotion showed more self-efficacy than people high on prevention. Although the interaction between RFI and the coaching intervention failed to reach the significance level of 5%, simple slopes indicate that promoters (*p* = .017) indicated higher self-efficacy when they received the promotion relative to the prevention intervention but for preventers (*p* = .760) the promotion or prevention intervention did not make a difference.

#### Goal-related motivation

For *intrinsic motivation*, there was a significant interaction between RFI and coaching intervention. Simple slopes showed that promoters (*p* = .054) indicated higher intrinsic motivation when they received the promotion relative to the prevention intervention but for preventers (*p* = .329) the promotion or prevention intervention did not make a difference. For *identified regulation*, there were no significant effects.

#### Goal commitment

The interaction between RFI and coaching intervention failed to reach the significance level of 5%. Simple slopes revealed that for promoters (*p* = .480) the promotion or prevention intervention did not make a difference but preventers (*p* = .090) indicated more goal commitment when they received the prevention relative to the promotion intervention. Thus, there is some evidence that regulatory focus moderates the relationship of the intervention and goal commitment with preventers showing stronger goal commitment when they received the prevention relative to the promotion intervention.

#### Implicit approach motivation

*We included the mean handedness quotient* as a covariate in our analyses. The results revealed a significant main effect of RFI, indicating that overall people high on promotion showed lower increase in approach motivation than people high on prevention. The interaction between RFI and coaching intervention for people’s approach motivation failed to reach the significance level of 5%. However, simple slopes revealed that for promoters (*p* = .660) the promotion or prevention intervention did not make a difference but preventers (*p* = .046) had an increase in approach motivation when they received the prevention relative to the promotion intervention.

#### Correction for multiple comparisons

As for our hypothesis H2, we test five dependent variables, we performed an alpha correction for multiple comparisons. Controlling for the family-wise error rate, we applied the Bonferroni correction with an alpha level of .05 and five tests. This results in a corrected alpha level of *p* = .001. Interpreting the findings with the corrected alpha level, all of the significant interactions of RFI with intervention disappear.

### Discussion

In Study 3a we tested the regulatory fit hypothesis in relation to different goal setting strategies in the action planning phase of a coaching. Although interaction effects between coaching intervention and people’s regulatory focus were not all *p* < .05, the results of this study provide some, although weak, empirical support for H2, i.e., the idea of the applicability of the regulatory fit effect regarding coaching techniques. Specifically, we found that for self-efficacy and intrinsic motivation, individuals with a more pronounced promotion focus benefit from a regulatory fit–they indicate higher self-efficacy and more intrinsic motivation when they receive a goal-setting intervention that focuses on promotion-related actions. When asked to record avoidance strategies during goal setting, perceived self-efficacy as well as intrinsic motivation decline. For individuals with a more pronounced prevention focus our results are rather equivocal, with no effect for self-efficacy and intrinsic motivation, only a marginal effect for goal commitment, but a significant effect for implicit approach motivation. Thus, preventers may benefit from a regulatory fit on an implicit level–they show more positive values on our measure for approach motivation when they receive an intervention that focuses on prevention-related actions. However, the missing results for preventers’ intrinsic motivation or self-efficacy again raise the question introduced earlier, as to whether coaching by its very nature better fits the motivational orientation of promoters, based on change and growth, than that of change-reluctant preventers.

Yet, following the regulatory fit hypothesis, we still believe that motivational congruency can compensate for the presumed disadvantages of a prevention focus. Some researchers emphasize the need for more follow-up assessments in coaching studies to obtain a more holistic picture of the effects and their durability [87, 88]. That is, for some clients, coaching benefits might only appear after a specific time required to cognitively process the intervention and its implications have elapsed. This led us to conduct another coaching study, measuring time-delay effects.

## Study 3b

In this study, we instructed participants to reflect upon goal-relevant personal resources, directed towards serving either growth or security needs and investigated immediate, as well as time-delayed regulatory fit effects. We hypothesized that promotion-focused coaching clients benefit more from identifying personal energy-givers and that prevention-focused coaching clients benefit more from identifying personal energy-takers.

### Method

#### Participants

Based on an a priori power analysis requiring a minimum sample size of *N* = 81 (see Study 3a), a total of 85 German-speaking participants was recruited for this online study via university newsletters, social networks, or personal contact. The language of the survey was German. Three participants were eliminated due to neglecting to work on the coaching intervention. Participants were randomly assigned to the promotion (*n* = 42) or prevention coaching intervention (*n* = 43). There was an exploratory third condition, the prevention-plus-affirmation condition (*n* = 37), not part of the main analysis. For further information, please contact the first author. The participants’ age ranged from 18 to 53 years with *M*_age_ = 24.32 years (*SD* = 6.37) and a gender distribution of 70 females and 15 males. Of the 85 individuals participating in Stage 1 of the study, 57 returned to attend Stage 2 of the study, 1 week later. Students in the sample received course credit in exchange for their participation. Grubbs’ outlier testing led to the exclusion of one participant for the analysis of self-efficacy.

#### Procedure and measures

In Stage 1 of the study, participants’ regulatory focus and baseline measures of state self-esteem, self-efficacy, positive and negative affect (t1) were assessed. Then, we applied the respective version of the coaching intervention “energy-map”, which is used in coaching sessions to help clients reflect upon personal resources in different areas of their life [89]. In this application, areas are listed separately and include friends/social contacts, romantic relationships, self, leisure time, family, job/studies, and an additional area free of choice. Whereas the original version of the energy map aims to identify both “energy-givers” and “energy-takers” in the respective areas, for purposes of our study we deconstructed the map into two separate versions, which served as the underlying manipulation.

In the *promotion version* of the intervention, participants were asked to reflect upon and write down personal *energy-givers* in their areas of life (i.e., friends, partnership, self, leisure time, the family of origin, academic/professional context, and an open free-choice category). On a little side note, they were given a set of exemplary ideas on which aspects to focus on. Aside from nine shared aspects (e.g., goals, feelings, thoughts, tasks, activities, relationships, social contacts, beliefs, environment), there were also two promotion-specific aspects: performances and creations. In the *prevention version*, participants were asked to reflect upon and write down personal *energy-takers* in their areas of life. They were given the same set of shared aspects listed above and further, two unshared prevention-specific aspects to focus on: obligations and responsibilities.

Apart from these major differences, both versions of the coaching intervention shared the same underlying storyline, namely that becoming aware of their resources can support them in better implementing their goals. After working on the respective coaching intervention, participants filled out questionnaires assessing state self-esteem, self-efficacy, and positive and negative affect (t2) for a second time, before finishing Stage 1 of the study with a set of demographic questions. One week later, at Stage 2 of the study, state self-esteem, self-efficacy, and positive and negative affect were assessed for a third time (t3), together with satisfaction with coaching. For all variables assessed three times (t1, t2, t3), we built difference scores reflecting the change from t1 to t2 and from t1 to t3. All measures are described in detail in S1 Table.

#### Statistical analysis

**Fig 3:**
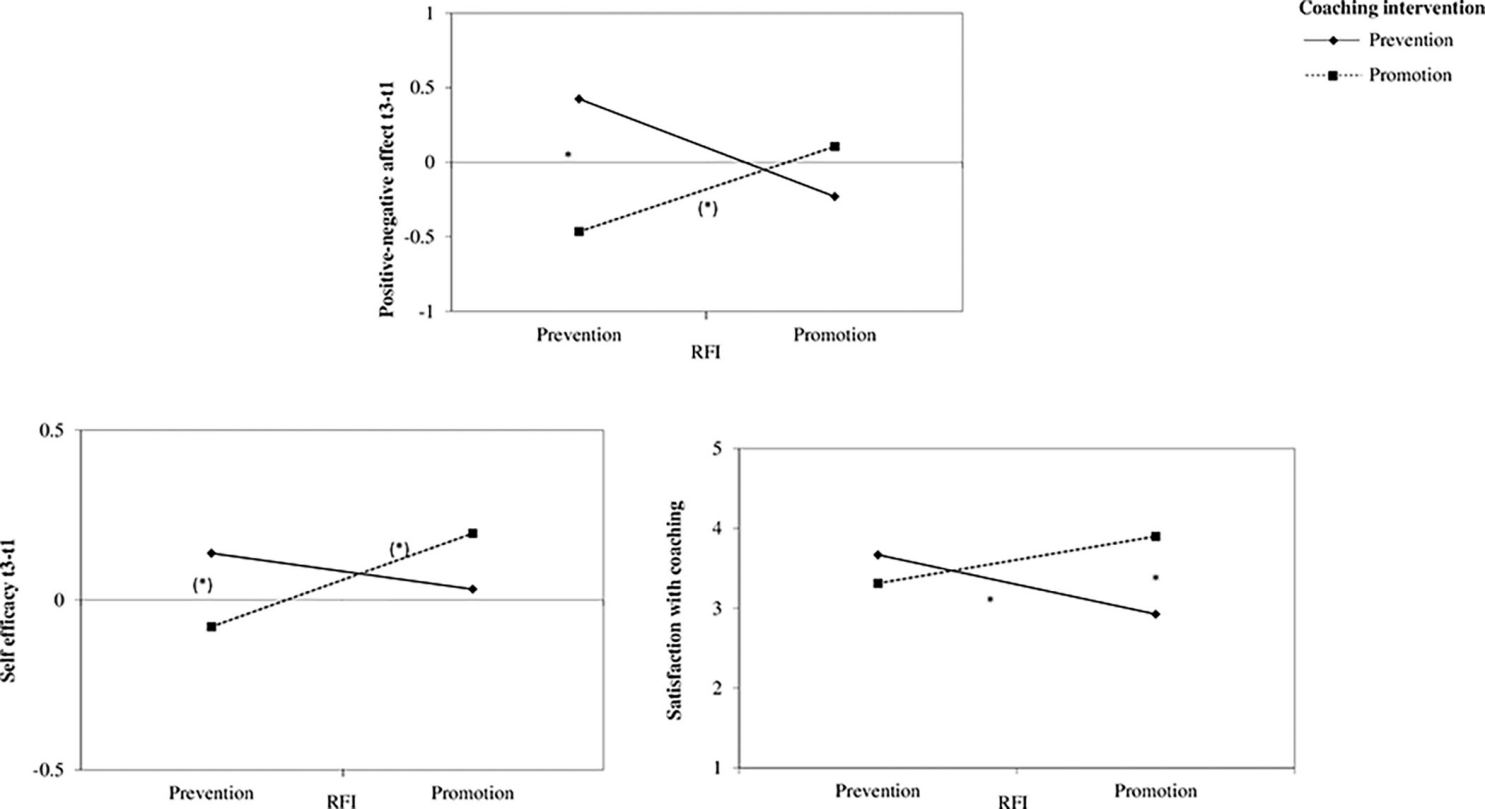
Interaction effects of RFI and coaching intervention on the dependent variables in Study 3b. * p < .05.

Statistical analyses were the same as in Study 3a. See Table 4 for the estimates and Fig 3 for the interaction effects.

**Table 4:** Estimates for RFI, the intervention and their interaction for Study 3b.

Variable	R^2^	Predictor	*b*	Lower 95% CI	Upper 95% CI	*SE*	*t*	*p*
Self-esteem t2-t1	.06		* *			* *		
		RFI	-0.07	-0.15	0.01	0.04	-1.73	.088
		**Intervention**	**0.17**	**0.01**	**0.33**	**0.08**	**2.15**	**.035**
	R^2^ change: .03	RFI x Intervention	0.11	-0.02	0.23	0.06	1.65	.103
Self-esteem t3-t1	.01							
		RFI	-0.03	-0.16	0.10	0.07	-0.47	.643
		Intervention	0.004	-0.30	0.31	0.15	0.03	.981
	R^2^ change < .001	RFI x Intervention	-0.004	-0.25	0.24	0.12	-0.04	.972
Self-efficacy t2-t1	.06							
		RFI	-0.002	-0.06	0.05	0.03	-0.07	.955
		Intervention	0.07	-0.03	0.18	0.05	1.43	.155
	R^2^ change: .03	RFI x Intervention	0.07	-0.02	0.15	0.04	1.63	.107
Self-efficacy t3-t1	.10							
		RFI	-0.05	-0.14	0.04	0.05	-1.09	.279
		Intervention	0.10	-0.11	0.30	0.10	0.96	.340
	R^2^ change: .08	**RFI x Intervention**	**0.18**	**0.02**	**0.34**	**0.08**	**2.19**	**.033**
Affect t2-t1	.04							
		RFI	-0.12	-0.35	0.11	0.11	-1.04	.300
		Intervention	0.36	-0.09	0.8	0.22	1.60	.113
	R^2^ change: .003	RFI x Intervention	0.09	-0.27	0.44	0.18	0.48	.635
Affect t3-t1	.10		** **	** **	** **	** **	** **	** **
		**RFI**	**-0.31**	**-0.65**	**0.04**	**0.17**	**-1.78**	**.081**
		Intervention	0.13	-0.67	0.93	0.40	0.32	.750
	R^2^ change: .06	**RFI x Intervention**	**0.58**	**-0.05**	**1.22**	**0.32**	**1.84**	**.071**
Satisfaction with coaching	.12		** **	** **	** **	** **	** **	** **
		**RFI**	**-0.35**	**-0.67**	**-0.04**	**0.16**	**-2.25**	**.029**
		**Intervention**	**0.75**	**0.02**	**1.48**	**0.36**	**2.07**	**.044**
	R^2^ change: .08	**RFI x Intervention**	**0.63**	**0.05**	**1.22**	**0.29**	**2.18**	**.034**

Significant effects at *p* < 0.05 and effects that reached a significance level of *p* < 0.10 are in bold. Coding of coaching intervention: 0 = prevention, 1 = promotion.

### Results

#### Change in self-esteem

For the change in *self-esteem from t1 to t2*, there was a significant main effect of the coaching intervention, indicating that overall, people working on the promotion intervention indicated more increase in self-esteem than people working on the prevention intervention. For the change in *self-esteem from t1 to t3*, there were no significant effects.

#### Change in self-efficacy

For the change in *self-efficacy from t1 to t2*, there were no significant effects. For the change in *self-efficacy from t1 to t3*, there was a significant interaction between RFI and the coaching intervention. Simple slopes showed that preventers (*p* = .050) showed more increase in self-efficacy when they received the prevention relative to the promotion intervention and participants who worked on the promotion intervention showed a more increase in self-efficacy when they were promoters than when they were preventers (*p* = .062).

#### Change in affect

For participants’ change in *affect from t1 to t2*, there were no significant effects. For participants’ change in *affect from t1 to t3* the main effect of RFI and the interaction between RFI and the coaching intervention failed to reach the significance level of 5%. Simple slopes indicated that preventers (*p* = .031) showed a higher increase in positive affect when they received the prevention relative to the promotion intervention and participants who worked on the promotion intervention reported a higher increase in positive affect when they were promoters than when they were preventers (*p* = .081).

#### Satisfaction with coaching

There was a significant main effect of RFI, revealing that overall, preventers indicated more satisfaction than promoters. There was also a significant main effect of the coaching intervention, indicating that overall, people working on the promotion intervention indicated more satisfaction than people working on the prevention intervention. Further, the interaction between RFI and coaching intervention was significant. Simple slopes showed that promoters (*p* = .037) indicated more satisfaction with coaching when they received the promotion relative to the prevention intervention and participants who worked on the prevention intervention reported more satisfaction with coaching when they were preventers than when they were promoters (*p* = .029).

#### Correction for multiple comparisons

As for our hypothesis H2, we test four dependent variables, we performed an alpha correction for multiple comparisons. Controlling for the family-wise error rate, we applied the Bonferroni correction with an alpha level of .05 and four tests. This results in a corrected alpha level of *p* = .013. Interpreting the findings with the corrected alpha level, all of the significant interactions of RFI with intervention disappear.

### Discussion

This study investigated how regulatory focus affects the success of coaching interventions aimed at managing one’s resources. As in Study 3a, we detected small regulatory fit effects. Interestingly, in this study, fit effects were observed only with a delay, i.e. one week after the working on the coaching intervention. There were no fit effects immediately after the intervention. Following a time-delayed assessment, our analyses revealed a regulatory fit effect for promoters’ satisfaction with coaching and preventers’ self-efficacy and their positive affect. One week after working on the coaching intervention, promoters reported more satisfaction when they had received the promotion compared to the prevention intervention, and preventers’ showed a higher increase in self-efficacy and positive affect when they had received the prevention compared to the promotion intervention. When preventers had received the promotion intervention, their self-efficacy even decreased and they experienced an increase in negative affect compared to the baseline measure before the coaching intervention.

However, regulatory fit effects found in this study should be interpreted with caution. First, one week after working on the coaching intervention, the follow-up questionnaire was only filled out by 57 participants. Second, a corrected alpha level leads to a disappearance of all of the significant interactions. Although there are certainly limitations of the current study, it has implications for research on coaching. First, coaching benefits do not always seem to show up immediately after a coaching intervention but may appear with a time delay. Second, our study supports the claim of some coaching researchers calling for more follow-up assessments when investigating the effectiveness of coaching interventions [87, 88]. In sum, a coaching intervention aimed at energy givers or takers may have potential but a more powered study may provide clarity regarding the effects of it for promoters and preventers.

## Study 3c

Whereas Study 3a revealed regulatory fit effects primarily for promoters and Study 3b revealed additional regulatory fit effects for preventers with a time delay, Study 3c sought to test regulatory fit with no time delay following the intervention. Although maintaining a parallel study design, we implemented some changes in Study 3c. Apart from motivation-based orientation towards growth or security, the two self-regulatory systems also differ according to the level of abstraction at which mental representations are construed. Whereas promotion focus is more strongly associated with processing information at an abstract level (e.g., focusing on the “why” of an action) and construing future events on a more distal temporal perspective, a prevention focus is associated with processing information on a concrete level (e.g., focusing on the “how” of an action) and construing future events on a more proximal temporal perspective [72, 90, 91]. Therefore, if the regulatory focus is congruent with a manipulated level of construal (i.e., if there is a regulatory construal fit), processing fluency, task engagement, and favorable attitudes towards a message will be increased [72, 91]. For instance, if one wants to heighten people’s engagement in sports, individuals with a promotion focus are more likely to be persuaded by a message that is formulated more abstractly by stating general benefits of engaging in sports, whereas individuals with a prevention focus are comparably more persuaded by a message formulated more concretely by emphasizing the specific number of calories burnt when exercising [92]. In other words, a match between people’s regulatory focus (promotion vs. prevention) and the construal level of a message (abstract vs. concrete) increases behavioral intention. Additionally, individuals not only differ in the way they encode information but also use different linguistic registers when referring to their goals. That is, a promotion focus involves describing a goal in abstract, more global strategic formulations (e.g., being good, friendly, or available), whereas a prevention focus involves the use of concrete, more specific terms in describing a goal [e.g., keeping in touch, phoning, visiting frequently; 92]. Accordingly, in this study, we built upon findings on regulatory construal fit effects [72] and added a cognitive component by asking participants to reflect upon a previously set personal goal in abstract terms (high construal level) or concrete terms (low construal level).

Based on H2, we hypothesized that promotion-focused clients benefit from abstract and prevention-focused clients benefit from concrete coaching tasks. We further hypothesized that regulatory fit effects between construal level and one’s regulatory focus are mediated by the experience of value from fit (H4).

### Method

#### Participants

To determine the minimum sample size required to test our mediation hypothesis (H4), we used the guideline values by Fritz and MacKinnon [93, Table 3]. The table indicated that for percentile bootstrapping, detecting an effect between small and medium (HH) and achieving a power of 80%, the required sample size was *N* = 162. We obtained a sample size of 192 German-speaking participants who were recruited for this online study via university newsletters, social networks, or personal contact. Three participants were excluded due to inadequately executing the coaching intervention. Thus, data of 189 participants (152 females, 37 males) aged from 17 to 58 years with *M*_age_ = 24.59 (*SD* = 6.71) were included in the statistical analyses. Regarding profession, 163 of them were students, 21 were employed, 4 were unemployed, and one did not indicate profession. Participating students in the sample received course credit, and each participant was invited to enter a lottery to win a 25€ Amazon coupon. Participants were randomly assigned to either the abstract (*n* = 106) or concrete coaching intervention (*n* = 83).

#### Procedure and measures

After assessing regulatory focus, participants were asked to write down an important academic or professional career goal they would like to achieve in the next 4 months. Then, they indicated where they would like to be regarding their goal attainment after these 4 months and where they see themselves at the moment (on a scale from 1 [*not attained at all*] to 10 [*fully attained*]). This was followed by the LBT to assess implicit approach motivation (see Study 1 and 3a). Next, participants received the coaching intervention (i.e., the experimental manipulation), which was adapted from the “how/why” task [94]. The “abstract” group was asked to think about why they would like to achieve their goal. This was followed by three why-type questions, each referring to the previously given answer. The “concrete” group was asked to think about how they would like to achieve their goal. This was followed by three how-type questions, each referring to the previously given answer. Please see S1 File for the exact coaching interventions. After the coaching intervention, participants completed another LBT and filled out a questionnaire assessing goal attainment, self-efficacy, goal-related motivation, goal commitment, value from fit, and indicated their intended time of goal initiation and some demographic information. All measures are described in detail in S1 Table.

#### Statistical analysis

To test whether value from fit mediates the relationship between intervention and coaching outcomes, we used moderated mediation analysis (model 7) of the SPSS macro PROCESS by Hayes [84] with the coaching intervention (dummy coded: concrete task = 0, abstract task = 1) as independent variable, RFI as moderator variable and value from fit as mediator variable, and the coaching outcomes goal attainment, self-efficacy, intrinsic motivation, identified regulation, goal commitment, and intended time of goal initiation as dependent variables. We used a 95% bias-corrected bootstrap confidence interval and 10,000 bootstrap samples.

### Results

First, we report the moderation analyses on value from fit with coaching intervention (coded with concrete = 0, abstract = 1) as an independent variable and people’s RFI as a moderator variable. We used SPSS macro PROCESS with a 95% bias-corrected bootstrap confidence interval (95% BCCI) and 10,000 bootstrap samples [84, model 1]. For significant results, follow-up simple slope analyses are investigated using the conditional effect of the intervention condition on the dependent variables for promoters (+1 SD of RFI, i.e., dominant promotion focus) and preventers (–1 SD of RFI, i.e., dominant prevention focus).

#### Value from fit

**Fig 4:**
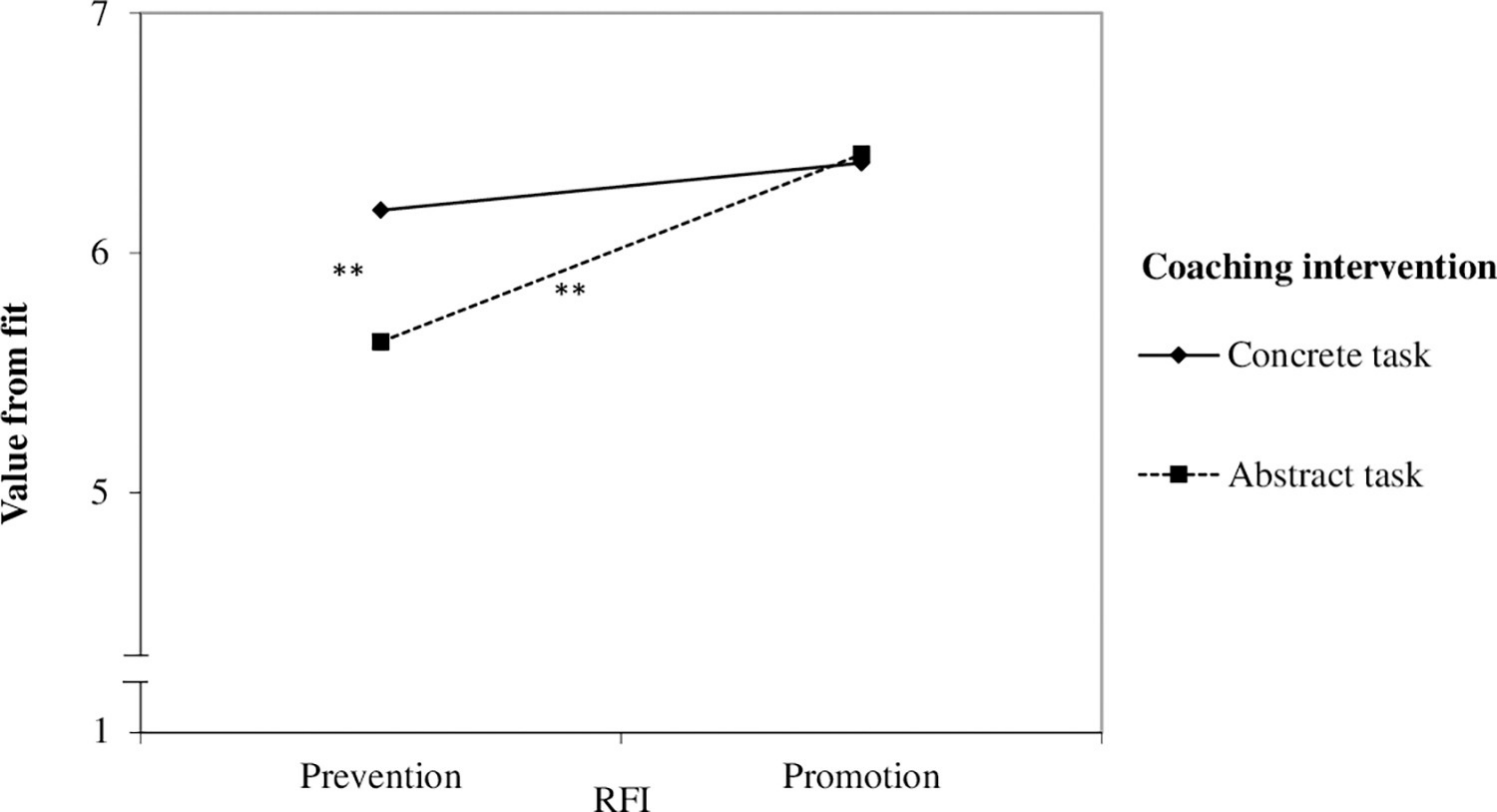
Interaction effects of RFI and coaching intervention on value from fit in Study 3c. ** p < .01.

The moderation analysis showed no significant main effect of RFI on value from fit, *b* = 0.08, SE = 0.05, 95% CI [-0.03; -0.19], *t*(185) = 1.43, *p* = .154. However, there was a significant main effect of the coaching intervention, *b* = -0.50, SE = 0.13, 95% CI [-0.73; -0.21], *t*(185) = -3.60, *p* < .001, indicating that participants who worked on the abstract task felt more right than participants who worked on the concrete task. This main effect was qualified by a significant interaction between coaching intervention and RFI, *b =* 0.23, SE = 0.08, 95% CI [0.06; 0.40], *t*(185) = 2.72, *p* = .007 (interaction model: R^2^ = .15; R^2^ change due to interaction = .03). Simple slopes showed no significant relationship between the coaching intervention and value from fit for promoters, *b =* 0.03, SE = 0.15, 95% CI [–0.26; 0.33], *t*(185) = 0.22, *p* = .824, but a significant negative relationship between the coaching intervention and value from fit for preventers, *b =* –0.55, SE = 0.15, 95% CI [–0.84; –0.25], *t*(185) = –3.67, *p* < .001. This finding indicates that preventers assigned more value to the coaching when they received the concrete relative to the abstract intervention. Thus, preventers but not promoters derived value from regulatory construal fit (Fig 4).

#### Moderated mediation

We predicted that the type of coaching intervention (abstract vs. concrete) would predict approach motivation, goal attainment, self-efficacy, intrinsic motivation, identified regulation, goal commitment, and intended time of goal initiation via value from fit with RFI moderating the effect. The findings of the analyses are summarized in Table 5 and indicate that the dominant regulatory focus moderated the relation between the coaching intervention and most coaching outcomes due to an increased value of the coaching. The moderated mediation showed small to medium significant effects for self-efficacy, intrinsic motivation, identified regulation, goal commitment, and intended time of goal initiation, *p*s < .05, but no significant effects for implicit approach motivation and goal attainment. Analyses of conditional indirect effects revealed that the regulatory construal fit effects (mediated by value from fit) were present for preventers but not for promoters. These results indicate that preventers seem to have an increased experience of feeling right about their goal after working on the *how* task relative to the why task. This regulatory construal fit in turn increased intrinsic motivation regarding their goal-related activities, goal commitment, and leads preventers to start realizing their goals. The moderated mediation effect was not found for promoters, probably because promoters had already relatively high levels of motivation.

**Table 5:** Moderated mediations for Study 3c.

Coaching outcomes	R^2^	Effects		Estimate	*b*	*SE*	[95% CI]
Implicit approach motivation	.001	Effect of intervention on outcome			-0.09	0.33	[-0.74; 0.56]
** **	** **	Effect of value from fit on outcome			-0.04	0.21	[-0.46; 0.37]
** **	** **	Moderated mediation effect		-0.01		0.05	[-0.13; 0.08]
		Conditional indirect effects	Promotion focus (+1 SD)		0.00	0.04	[–0.08; 0.07]
			Prevention focus (–1 SD)		0.02	0.12	[–0.19; 0.30]
Goal attainment	.01	Effect of intervention on outcome			-0.05	0.35	[-0.74; 0.65]
** **	** **	Effect of value from fit on outcome			0.22	0.23	[-0.23; 0.67]
** **	** **	Moderated mediation effect		0.05		0.06	[-0.04; 0.18]
		Conditional indirect effects	Promotion focus (+1 SD)		0.02	0.05	[–0.08; 0.14]
			Prevention focus (–1 SD)		–0.12	0.12	[–0.39; 0.10]
Self-efficacy	.05	Effect of intervention on outcome			0.18	0.13	[–0.08; 0.43]
** **	** **	**Effect of value from fit on outcome**		** **	**0.26**	**0.08**	**[0.09; 0.42]**
** **	** **	**Moderated mediation effect**		**0.06**	** **	**0.03**	**[0.01; 0.12]**
		Conditional indirect effects	Promotion focus (+1 SD)		0.01	0.04	[–0.07; 0.09]
			**Prevention focus (–1 SD)**	** **	**–0.14**	**0.06**	**[–0.27; –0.03]**
Intrinsic motivation	.07	Effect of intervention on outcome			0.32	0.21	[–0.09; 0.73]
		**Effect of value from fit on outcome**		** **	**0.48**	**0.13**	**[0.22; 0.74]**
		**Moderated mediation effect**		**0.11**	** **	**0.05**	**[0.01; 0.22]**
		Conditional indirect effects	Promotion focus (+1 SD)		0.02	0.07	[–0.13; 0.16]
			**Prevention focus (–1 SD)**	** **	**–0.26**	**0.11**	**[–0.49; –0.08]**
Identified regulation	.12	Effect of intervention on outcome			0.06	0.16	[–0.26; 0.37]
		**Effect of value from fit on outcome**		** **	**0.51**	**0.10**	**[0.31; 0.71]**
		**Moderated mediation effect**		**0.12**	** **	**0.06**	**[0.01; 0.24]**
		Conditional indirect effects	Promotion focus (+1 SD)		0.02	0.08	[–0.14; 0.18]
			**Prevention focus (–1 SD)**	** **	**–0.28**	**0.11**	**[–0.51; –0.09]**
Goal commitment	.21	Effect of intervention on outcome			0.07	0.11	[–0.14; 0.28]
		**Effect of value from fit on outcome**		** **	**0.48**	**0.07**	**[0.34; 0.61]**
		**Moderated mediation effect**		**0.11**	** **	**0.05**	**[0.02; 0.20]**
		Conditional indirect effects	Promotion focus (+1 SD)		0.02	0.07	[–0.13; 0.16]
			**Prevention focus (–1 SD)**	** **	**–0.26**	**0.08**	**[–0.42; –0.10]**
Intended time of goal initiation	.07	Effect of intervention on outcome			0.01	0.16	[–0.31; 0.32]
		**Effect of value from fit on outcome**		** **	**–0.27**	**0.10**	**[–0.47; –0.06]**
		**Moderated mediation effect**		**–0.06**	** **	**0.05**	**[–0.16; –0.001]**
		Conditional indirect effects	Promotion focus (+1 SD)		–0.01	0.04	[–0.12; 0.06]
			**Prevention focus (–1 SD)**	** **	**0.14**	**0.08**	**[0.01; 0.32]**

Exact p-value is not provided in this table. Bold text (CI not including 0) indicates a significant effect at *p* < .05

^a^ coding of intervention: 0 = concrete how task; 1 = abstract why task

#### Correction for multiple comparisons

As for our moderated mediations, we test six dependent variables, i.e., self-efficacy, intrinsic motivation, identified regulation, goal commitment, implicit approach motivation, and intended time of goal initiation, we performed an alpha correction for multiple comparisons. Controlling for the family-wise error rate, we applied the Bonferroni correction with an alpha level of .05 and six tests. This results in a corrected alpha level of *p* = .008. Interpreting the finding that the value from fit mediates the relationship of RFI and intervention on the dependent variables with the corrected alpha level, all effects, expect of intended time of goal initiation, remain.

### Discussion

In Study 3c we sought to extend regulatory fit findings from Studies 3a and 3b (H2), by adding a cognitive component that is related to regulatory focus (i.e., construal level). The findings provide support for regulatory construal fit in coaching. Additionally, we found that individuals derived value from fit and that this process variable positively affected coaching outcomes, providing support for H4. Particularly convincing were the results for individuals with a dominant prevention focus. Preventers seemed to assign a higher value to their goal and felt right about it when they reflect on how to achieve the goal instead of why. This experience seems to be a positive enhancer for coaching outcomes. As value from fit includes aspects like positive prospective and retrospective feelings about a goal, these results appear especially important, showing that regulatory construal fit might support preventers in shifting their focus from avoiding negative affective states to approaching positive ones. In other words, regulatory construal fit may allow preventers to open up for personal growth and change, as their need for security is perceived as unthreatened.

For promoters, results revealed a different picture. Whether or not they felt right about their goal was independent of a regulatory fit experience. Consequently, feeling right from fit did not show the positive enhancement attributes for other coaching outcomes, as it did for preventers. In sum, there were significant positive correlations for dominant regulatory focus with coaching outcomes (see S2 Table). These findings indicate that aside from more feeling right, promoters showed higher levels of self-efficacy, motivation, and goal commitment than preventers to begin with. This suggests that a promotion focus by itself is a good predictor of positive coaching outcomes when working with cognitive interventions. This result also provides support for H1 hypothesizing that clients with a higher promotion than prevention focus report more coaching success than clients with a higher prevention than promotion focus. The level of mental abstraction in a coaching intervention seems to matter significantly more for preventers than it does for promoters. This finding supports targeted matching of coaching interventions on not only a motivational (growth vs. security) but also a cognitive level (abstract vs. concrete thinking), particularly when working with preventers. Promoters on the other hand seemed to benefit from operationalizing their goal in both an abstract and concrete manner.

This study makes several important contributions to research on regulatory focus. First, it extends findings from our previous studies by showing regulatory fit effects for preventers that are not only time-delayed (as in Study 3b) but also appear immediately after a coaching intervention. Second, we show evidence for regulatory construal fit in the coaching context. Similar to previous studies on regulatory fit [58, 72, 95], value from fit has shown to be an important underlying process variable of the fit effect, transferring the heightened value of the current experience to other outcome variables.

## Studies 4a and 4b –Interpersonal fit between client and coach

In Study 4a and 4b, we aim to test regulatory fit effects arising from interpersonal fit in coaching, i.e., the fit between the client’s and the coach’s regulatory focus. In Study 4a we used a correlational within-design. Participants were given the descriptions of a promotion- and a prevention-oriented coach and indicated their satisfaction with and their trust in the two coaches. In Study 4b we had videotaped real coaching sessions that we analyzed according to individuals’ regulatory focus. Here, real coaching clients indicated their coaching progress immediately and their goal attainment one month after the coaching.

## Study 4a

In this study we not only test the hypothesis regarding interpersonal fit (H3) but also the hypothesis that coaching in general appeals more to promotion- than to prevention-oriented clients (H1).

### Method

#### Participants, procedure, and measures

Forty-one German-speaking participants (30 females and 11 males) who were interested in the consulting format coaching were recruited for a paper-pencil study via personal contacts. Participants’ age ranged from 18 to 50 years with *M*_age_ = 25.42 years (*SD* = 7.43). After filling out a measure for the regulatory focus trait questionnaire, participants were instructed to imagine that the company they worked for offered coaching for personnel development. Then, participants read a short description of what coaching is and imagined that they could participate in such a coaching. Therefore, two coaches provided their services. Participants imagined that they could choose between these two coaches. The coaches–a promotion- and a prevention coach–were described and participants were asked to indicate how satisfied they would be with the coaches and how much they would trust the coaches. The order of the two descriptions was randomized. For the descriptions of the two coaches, please see S1 File. Participants finished the survey by indicating their gender and age.

Since we did not perform an a-priori power analysis for this study, we ran a sensitivity analysis using G*Power [80]. With our sample size of *N* = 41, we were able to detect an effect size of *r* = .38 with a power of .80, *r* = .40 with a power of .85, *r* = .44 with a power of .90, and *r* = .48 with a power of .95, indicating that the study had sufficient power to detect the found correlations between RFI and satisfaction with the promotion coach (*r* = .51) and between RFI and satisfaction with the prevention coach (*r* = -.65), but was slightly underpowered for the found correlations between RFI and general trust in the promotion coach (*r* = .36), and RFI and trust in the promotion coach’s benevolence (*r* = .37) and integrity (*r* = .31).

### Results

For means, standard deviations, and correlations see Table 6.

**Table 6:** Means, standard deviations, and correlations for Study 4a.

Variable	M	SD	1	2	3	4	5	6	7	8	9	10	11	12	13	14	15	16	17
1. RFI (Promotion-Prevention)	1.30	1.62	-																
2. Promotion	5.27	0.80	.71**	-															
3. Prevention	3.98	1.19	-.88**	-.28(*)	-														
4. Satisfaction (both coaches)	3.98	0.41	-0.16	0.07	0.26	-													
5. Satisfaction (Promotion coach)	4.29	0.71	.51**	.46**	-.39*	.50**	-												
6. Satisfaction (Prevention coach)	3.66	0.77	-.65**	-.35*	.64**	.60**	-.40*	-											
7. General trust (both coaches)	3.83	0.56	.24	.15	-.23	.31*	.47**	.11	-										
8. General trust (Promotion coach)	3.68	0.74	.36*	.25	-.32*	0.20	.63**	-.37*	.88**	-									
9. General trust (Prevention coach)	3.99	0.58	.01	-.04	-.04	.35*	0.11	0.26	.80**	.42**	-								
10. Trust ability (both coaches)	3.82	0.66	.21	.12	-.20	.30(*)	.45**	-.10	.92**	.83**	.71**	-							
11. Trust ability (Promotion coach)	3.57	0.91	.22	.19	-.17	.23	.58**	-.29(*)	.76**	.84**	.39*	.85**	-						
12. Trust ability (Prevention coach)	4.07	0.73	.10	-.02	-.15	.25	.08	.19	.72**	.46**	.79**	.75**	.29	-					
13. Trust benevolence (both coaches)	3.78	0.57	.13	.09	-.11	.36*	.37*	.04	.86**	.73*	.72**	.77**	.64**	.59**	-				
14. Trust benevolence (Promotion coach)	3.82	0.95	.37*	.27(*)	-.32*	.11	.52**	-.37*	.69**	.85**	.24	.63**	.56**	.43**	.70**	-			
15. Trust benevolence (Prevention coach)	3.74	0.83	-.25	-.18	.22	.37*	-.10	.48**	.38*	.03	.71**	.33*	.23	.31*	.57**	-.20	-		
16. Trust integrity (both coaches)	3.89	0.69	.29(*)	.17	-.28(*)	.17	.42**	-.21	.84**	.75**	.67**	.64**	.50**	.53**	.52**	.50**	.14	-	
17. Trust integrity (Promotion coach)	3.65	0.82	.31*	.17	-.31*	.15	.44**	-.25	.76**	.80**	.44**	.59**	.51**	.43**	.47**	.52**	.04	.89**	-
18. Trust integrity (Prevention coach)	4.15	0.75	.19	.13	-.17	.15	.28(*)	-.11	.72**	.50**	.76**	.54**	.37*	.52**	.45**	.34*	.22	.87**	.55**

(*) *p* < .10

* *p* < .05

** *p* < .01

To test H1, hypothesizing that a higher promotion than prevention focus is associated with a better evaluation of coaches in general, we averaged the responses for the scales (satisfaction and trust) over the promotion and the prevention coach. The correlation between RFI and satisfaction was not significant and the correlations between RFI and trust did not reach the significance level of 5%, indicating that a higher promotion than prevention focus of participants does not automatically mean a better evaluation of coaches (Table 6). Summarized, these findings do not provide support for H1.

For the fit-hypothesis H2, RFI shows significant positive correlations with the satisfaction with the promotion coach, *r*(39) = .51, *p* < .001, general trust in the promotion coach, *r*(39) = .36, *p* = .020, trust in the promotion coach’s benevolence, *r*(39) = .37, *p* = .017, and integrity, *r*(39) = .31, *p* = .048. RFI shows a significant negative correlation with satisfaction with the prevention coach, *r*(39) = -.65, *p* < .001. It seems that individuals with a more pronounced promotion focus are more satisfied with the promotion coach and individuals with a more pronounced prevention focus are more satisfied with a prevention coach. Although the other correlations of RFI with the prevention coach are not *p* < .05, they are lower than the correlations with the promotion coach or even negative (Table 6). The Olkin’s z comparisons between the correlations of RFI with the promotion coach and RFI with the prevention coach show significant differences on satisfaction, *z* = 7.14, *p* = .001, general trust, 2.34, *p* = .010, and trust in the coach’s benevolence, *z* = 2.90, *p* = .002. This means that higher scores on RFI (i.e., higher promotion than prevention focus) are associated with more satisfaction with the promotion coach, less satisfaction with the prevention coach, and more trust in the promotion coach, and vice versa for lower scores on RFI. There were no significant differences on trust in the coach’s ability or integrity, *p*_*s*_ ≤ .188.

Looking at the correlations of the separate promotion and prevention scores with the dependent variables, a high promotion focus is positively correlated with satisfaction with the promotion coach and negatively correlated with satisfaction with the prevention coach. A high prevention focus is positively correlated with satisfaction with the prevention coach and negatively correlated with satisfaction with the promotion coach and trust in the promotion coach.

#### Correction for multiple comparisons

As for our hypothesis H3, we test two dependent variables, i.e., satisfaction and trust, we performed an alpha correction for multiple comparisons. Controlling for the family-wise error rate, we applied the Bonferroni correction with an alpha level of .05 and two tests. This results in a corrected alpha level of *p* = .025. Interpreting the findings with the corrected alpha level, the significant correlations regarding satisfaction and general trust remain.

### Discussion

Summarized, potential promotion-focused coaching clients did not indicate more satisfaction with or trust in their coach than prevention-focused clients, not providing support for H1. Considering individuals’ regulatory focus, there were small fit effects for satisfaction as well as for general trust, and the subscales trust in the coach’s benevolence and integrity, thus providing support for H3. Specifically, a higher promotion than prevention focus was associated with satisfaction with and trust in the promotion coach and low satisfaction with the prevention coach. Vice versa, a higher prevention than promotion focus was associated with satisfaction with the prevention coach and low satisfaction with and low trust in the promotion coach. Of course, a vignette-based study should certainly not be transferred uncritically to actual coaching recommendations. We address this issue in the general discussion.

## Study 4b

In this study, we assessed regulatory focus in an implicit way. Regulatory focus is a construct usually lacking people’s full awareness and thus, questionnaires may not always provide accurate assessment of the regulatory focus. Thus, implicit methods may provide an advantage over explicit methods. Such an implicit method is the analysis of language which can be affected by individuals’ regulatory focus. Studies have for example used a word-fragment completion task in which participants could form promotion-oriented, prevention-oriented, or neutral words [96]. There is also a list of promotion and prevention words that can be used to analyze written or spoken text and thus, determine one’s regulatory focus [97]. In the current study, we had videos of real coaching processes and employed an artificial intelligence tool to analyze the promotion- or prevention-language of coaches and their clients.

### Method

#### Participants and procedure

In cooperation with the Freiburg Institute, 90 videos of real one-to-one coaching sessions were analyzed. Based on the power analysis in Study 3a, the sample was adequate to test our fit-hypothesis (H3). The sessions lasted between 25 and 95 minutes and were recorded with two to three cameras (one frontally to the coach and one frontally to the client) and two microphones. From the 90 coaches (57 female, 33 male) 33 coaches just started with a coaching training (novices), 36 were in the middle of a coaching training (intermediates), and 21 already finished a coaching training and had some experience with coaching (experts). The novices and intermediates performed their coaching sessions within the coaching training. Before the coaching session, clients wrote down up to three goals for the following month and indicated the importance of these goals. After the coaching session, clients answered a questionnaire assessing their subjective coaching progress. One month after the coaching, clients indicated their goal attainment. As three clients did not fill out the questionnaire, we performed our statistical analyses with a final sample of *N* = 87 coaching dyads.

#### Measures

*Regulatory focus*. The coaching sessions were literally transcribed based on Dresing and Pehl [98] and embedded into the tool PRECIRE® [99]. PRECIRE® breaks down the spoken language into its linguistic features and generates z-standardized values. From 80 linguistic features generated by PRECIRE®, we chose those associated with a promotion (e.g., successful, performance) and prevention focus (e.g., rules, stress) in the regulatory focus literature and performed a factor analysis examining the factor loadings. The factor analysis revealed 6 features for promotion focus and 8 features for the prevention-oriented language. For coach and client, we built separate promotion- and prevention scores. We built two regulatory focus indices (RFI, i.e., promotion minus prevention score), one for the coach and one for the client. Positive values indicate a relatively higher promotion than prevention language. The other measures are described in detail in S1 Table.

#### Statistical analysis

**Fig 5:**
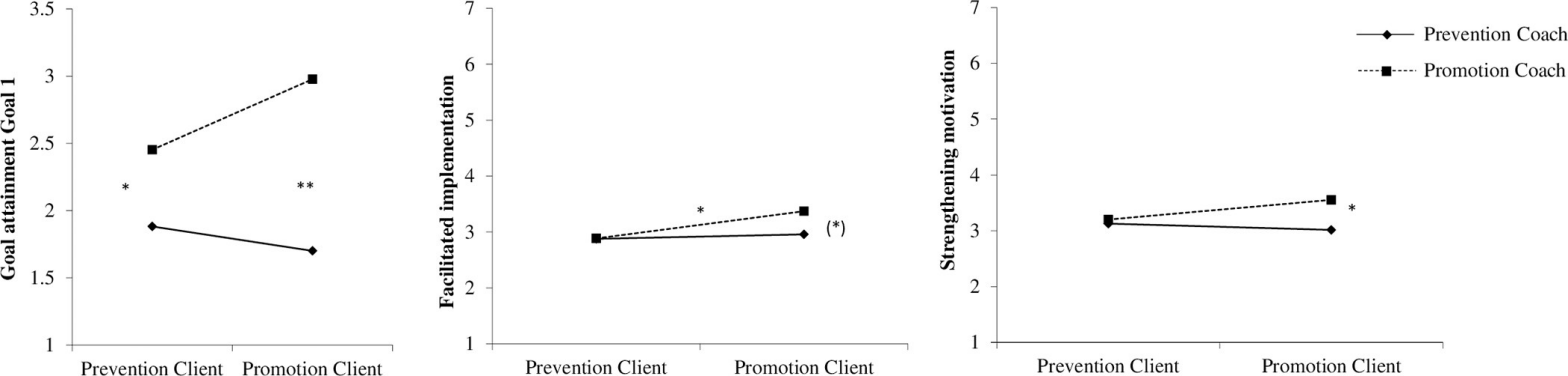
Interaction effects of the coach’s and client’s promotion- vs. prevention-oriented language on the dependent variables in Study 4b. (*) *p* < .10; * *p* < .05; ** *p* < .01.

Statistical analyses were the same as in Studies 3a and 3b. See Fig 5 for the interaction effects.

### Results

#### Goal attainment

There was a significant main effect for the coach’s language, *b =* 0.08, SE = 0.02, 95% CI [0.04; 0.12], *t*(78) = 3.76, *p* < .001, indicating that client had a higher goal attainment when the coach had a promotion-oriented relative to a prevention-oriented language. The interaction between the coach’s and client’s language was also significant, *b =* 0.01, SE = 0.002, 95% CI [0.001; 0.009], *t*(78) = 2.44, *p* = .017 (interaction model: R^2^ = .20; R^2^ change due to interaction = .06). Simple slopes showed a significant positive relationship between the coach language and goal attainment for clients with a promotion-oriented language, *b =* 0.10, SE = 0.03, 95% CI [0.05; 0.16], *t*(78) = 3.97, *p* < .001, and a significant positive relationship between the coach language and goal attainment for clients with a prevention-oriented language, *b =* 0.05, SE = 0.02, 95% CI [0.01; 0.09], *t*(78) = 2.52, *p* = .014. This indicates that clients using a promotion as well as prevention-oriented language have more goal attainment when they were coached by a coach using a promotion-oriented compared to a prevention-oriented language.

#### Coaching progress

For *enhanced understanding*, there was no significant interaction between the coach’s and client’s language, *b =* -0.001, SE = 0.001, 95% CI [-0.003; 0.001], *t*(83) = -0.76, *p* = .452 (interaction model: R^2^ = .02; R^2^ change due to interaction = .01). For *strengthened motivation*, the interaction between the coach’s and client’s language was significant, *b =* 0.003, SE = 0.001, 95% CI [0.00; 0.006], *t*(83) = 2.16, *p* = .034 (interaction model: R^2^ = .07; R^2^ change due to interaction = .05). Simple slopes showed a significant positive relationship between the coach language and goal attainment for clients with a promotion-oriented language, *b =* 0.04, SE = 0.02, 95% CI [0.003; 0.08], *t*(83) = 2.13, *p* = .036, but not for clients with a prevention-oriented language, *b =* 0.007, SE = 0.01, 95% CI [–0.02; 0.04], *t*(83) = 0.49, *p* = .627. For *facilitated implementation*, the interaction between the coach’s and client’s language reached exactly the significance level of 5%, *b =* 0.003, SE = 0.001, 95% CI [0.00; 0.01], *t*(82) = 1.99, *p* = .050 (interaction model: R^2^ = .08; R^2^ change due to interaction = .05). Simple slopes showed a marginal significant positive relationship between the coach language and goal attainment for clients with a promotion-oriented language, *b =* 0.03, SE = 0.02, 95% CI [-0.01; 0.07], *t*(82) = 1.71, *p* = .091, but no significant relationship for clients with a prevention-oriented language, *b =* 0.001, SE = 0.01, 95% CI [–0.03; 0.03], *t*(51) = 0.09, *p* = .928. This indicates that clients using a promotion-oriented language benefitted more from a coach using a promotion-oriented language relative to a prevention-oriented language but for clients with a prevention-oriented language, the language of the coach did not make a difference.

#### Correction for multiple comparisons

As for our hypothesis H3, we test four dependent variables, we performed an alpha correction for multiple comparisons. Controlling for the family-wise error rate, we applied the Bonferroni correction with an alpha level of .05 and four tests. This results in a corrected alpha level of *p* = .013. Interpreting the findings with the corrected alpha level, all of the significant interactions of RFI with intervention disappear.

### Discussion

Summarized, we found that promotion- as well as prevention-oriented clients indicated more goal attainment when they were coached by a promotion-oriented coach compared to a prevention-oriented coach. Testing H3, hypothesizing that a fit between the client’s and coach’s regulatory focus positively affects coaching success, we found a promotion but not a prevention fit for people’s coaching progress. Thus, there was no fit effect for clients with a prevention-oriented language and it seems that a coach with a promotion-oriented language is even more beneficial for them. These results partly support H3.

Although our effects are small and even disappear after alpha correction, the findings are in line with research on interpersonal fit [66] showing that the fit effects on goal-related motivation only appeared for promotion-oriented individuals but not for prevention-oriented individuals. They explained their findings by arguing that promoters may perceive help from another person as an opportunity and thus, are more receptive to such help, especially when there are similarities between their goal approach and the goal approach that the interaction partner uses. Due to their global processing style, they easily detect the similarities. Preventers, on the other hand, have a local processing style. They focus on possible threats and thus, fail to recognize the benefits of a helping interaction partner.

## General discussion

The present article investigates the effect of clients’ regulatory focus in coaching. First, we tested the hypothesis that coaching generally appeals more to promotion-oriented than to prevention-oriented clients. Although the effects were small, Study 1 showed that clients with a dominant promotion focus indicated a higher goal attainment and more increase in implicit approach motivation than clients with a dominant prevention focus whereas no difference was found in explicit approach motivation. Second, we tested the hypothesis that a fit between the regulatory focus of the client and the regulatory focus of the coaching approach positively affect coaching success, such that promotion-focused coaching approaches are more beneficial for promotion-focused clients and prevention-focused coaching approaches are more beneficial for prevention-focused clients. Study 2 showed that a more pronounced promotion focus was associated with more satisfaction and more approach motivation regarding promotion-oriented coachings. Studies 3a, 3b, and 3c showed that interventions matching with the regulatory focus of a client increased coaching success. In all three studies, participants were asked to set a personal career or academic goal before working on either a promotion or a prevention version of an established coaching intervention. We found fit effects for promoters as well as for preventers on different coaching outcomes. In Study 3a, using a task to define one’s goal (SMART method), promoters indicated more self-efficacy and more intrinsic motivation when they worked on a promotion- compared to a prevention-oriented goal task and preventers had more implicit approach motivation and goal commitment when they worked on a prevention- compared to a promotion-oriented goal task. In Study 3b, using a task defining energy-givers or takers, promoters indicated more satisfaction with coaching when they worked on the promotion- compared to the prevention-version (energy-givers). Only preventers, on the other hand, indicated an increase in positive affect and self-efficacy one week after they had worked on the prevention- compared to the promotion-version (energy-takers). In Study 3c, reflecting on the why vs. the how of one’s goals, only preventers experienced an increased value from the fitting how-task which mediated the relationship between regulatory fit and self-efficacy, intrinsic motivation, identified regulation, goal commitment, and intended time of goal initiation. Our results support the idea that a stronger promotion rather than prevention focus is beneficial for coaching, regardless of whether the reflection on a goal is abstract or concrete. This is indicated by the correlations shown in S2 Table. It suggests that promoters might have an advantage in coaching while preventers wo assign more value to their goal could benefit from regulatory fit.

Third, we tested the hypothesis that interpersonal fit, i.e., a fit between the client’s and the coach’s regulatory focus has benefits. Study 4a used a vignette-approach and showed that promotion-oriented clients were more satisfied with and relied more on a promotion-oriented coach than on a prevention-oriented coach. Prevention-oriented clients were more satisfied with and relied more on a prevention-oriented coach than on a promotion-oriented coach. Study 4b used real coaching sessions and showed that promotion-oriented clients indicated more coaching progress when they were coached by a promotion-oriented compared to a prevention-oriented coach. For clients’ goal attainment, a promotion- compared to a prevention-oriented coach was more favorable for promotion- as well as for prevention-oriented clients.

### Integrative summary of the findings of all studies

**Fig 6:**
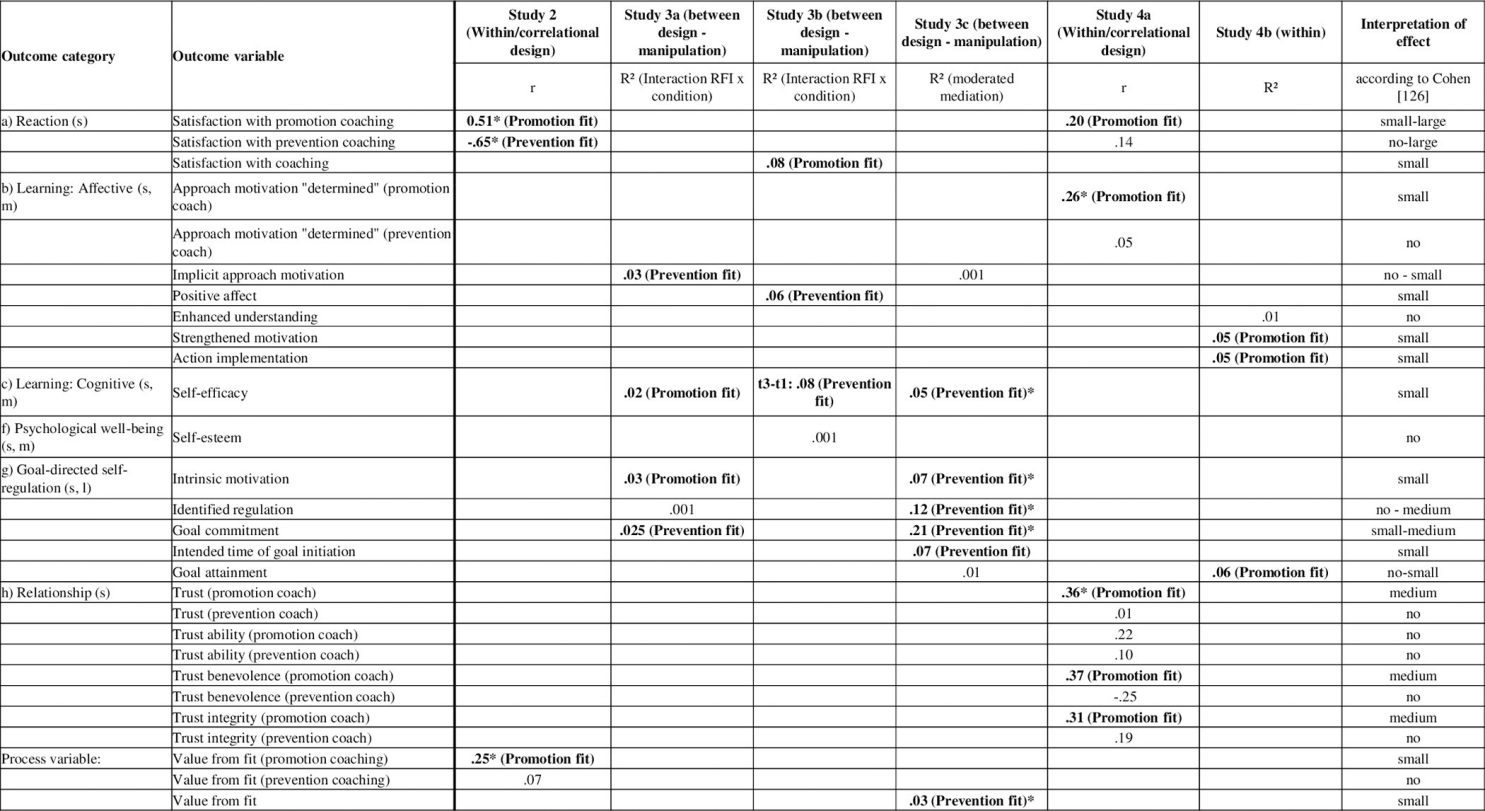
Summary of the findings of Studies 2-4b including the effect sizes and their interpretation according to Cohen [126], and comparison to the effects sizes reported in the coaching meta-analyses. The letters in brackets shows the effect size of the category reported in the coaching meta-analyses; s = small effects, m = medium effects, l = large effects; * effects remaining after alpha correction.

The studies provide evidence for regulatory fit in the coaching setting. In line with previous research on RFT in different areas we found that regulatory fit effects, albeit small to medium, are also present in coaching. Multiple studies assessing multiple variables relevant for coaching showed that the findings differ with regard to the interventions used and the variables that we looked at. Fig 6 summarizes the findings of all studies comparing the effect sizes. It also shows differences in the regulatory fit effects of preventers and promoters within the different studies. Preventers seem to benefit greatly from the exercise of making their goal concrete. Promoters do not necessarily need to engage in abstract thinking about their goal for it to be effective. This finding is compatible with research showing that when processing information or making decisions, promoters rely more on affect, whereas preventers rely more on reason [83, 100]. Therefore preventers are much more in thinking and brooding and a cognitive intervention as the one in Study 3c may thus be more beneficial for them. For the other interventions in Study 3a and 3b, both promoters and preventers show benefits: Promoters seem to be more satisfied when they are allowed to write down energy-givers; preventers experience more positive affect and more self-efficacy when they are allowed to write down energy-takers. When it comes to goal setting, preventers have more implicit approach motivation and more goal commitment when thinking about steps to avoid in order to reach a goal; promoters have more self-efficacy and intrinsic motivation when thinking about steps that are necessary to reach a goal.

For Study 1, testing our hypothesis that coaching appeals more to promoters than preventers, we found positive correlations of RFI with implicit but not explicit approach motivation. Since the two constructs did not even correlate with each other, they may reflect distinct constructs. While promotion and prevention orientation is a result of socialization, approach and avoidance motivation are biological constructs representing an impulse to move toward or away from something. This impulse can be a trait but can also be triggered by a situation [30]. Implicit approach motivation is usually measured using neuropsychological methods such as EEG. Explicit approach measures for example ask participants to indicate their approach-related feelings such as feeling capable, energized, or even angry [28, 101]. While approach motivation measured by EEG has been shown to be associated with trait and state approach motivation self-reports [e.g. 101], our study does not show a link between implicit and explicit approach motivation. One explanation may be that the LBT may not fully capture approach motivation assessed by EEG or that it measures something different [102]. Another explanation may be that approach processes lack individuals’ full awareness and thus, explicit and implicit measures reflect different findings. In our study, we asked participants to indicate their approach motivation regarding the coaching (“Right now, after the coaching session, I feel…” in Study 1, or “I am determined to participate in this coaching” in Study 2). This may reflect a different approach motivation than the more general approach motivation assessed by the LBT. The LBT may capture a general state of readiness to move toward, while the self-reported approach motivation we assessed may capture a general feeling of energization, capability, and determination.

### Strengths, limitations, and future research

Taken together, our results support previous findings on the positive effects of self-congruency on enhancing the experience of autonomy as well as on facilitating self-regulation and goal pursuit [8, 48, 103, 104]. Further, our studies contribute to a better understanding of RFT in several ways. First, regulatory fit effects are transferable to the coaching context. Second, fit effects do not always unfold immediately after an intervention, but also after a time delay. Third, regulatory construal fit effects may foster experiences of value from fit in coaching. Fourth and finally, fit effects can be found for promoters and preventers alike, though their prevalence seems to be dependent on the type of intervention, coaching outcome, and time frame. Thus, regulatory focus and fit should not be neglected in the area of coaching. It has valuable effects on various relevant coaching outcomes, such as self-efficacy, intrinsic motivation, goal commitment, satisfaction with coaching, or intended time of goal initiation.

A strength of the present line of research is that the studies used a mixed-methods approach. That is, to assess coaching outcome variables and regulatory focus, we used online- as well as paper-pencil questionnaires and, in Study 4b, we used clients’ spoken language. As we received regulatory fit effects for promotion- as well as for revention-oriented clients in most of the studies, a mixed-methods approach seems advantageous. However, Study 4b gives us a somewhat different picture. There were fit effects only for promotion-oriented but not for prevention-oriented clients. For goal attainment, promotion-oriented as well as prevention-oriented clients benefitted most from a promotion-oriented coach. This finding need further examination. Since goal attainment is the result of a successful promotion towards one’s goal, a possible explanation for this finding could be that promotion-focused coaches are more effective in eliciting, motivating, or reinforcing the respective behavior and skills in clients than prevention-focused coaches. At the beginning of a coaching process, however, a prevention-focused mode seems advisable for prevention-oriented clients to gain their trust and satisfaction. The findings of Study 4b are different from the other studies, which raises the question of the comparability of the studies. Here, we did not measure regulatory focus but used an artificial intelligence tool with predefined categories to find out the clients’ and coaches’ regulatory focus in language. In the factor analysis, some linguistic features would theoretically have been assigned more to a prevention factor, but they loaded onto the promotion factor and vice versa. An experiment introducing coaches to the theory of regulatory focus and giving them concrete instructions on how to use a promotion- vs. prevention-oriented style of speaking, could be better able to verify our idea of regulatory interpersonal fit in coaching.

In the present set of studies, we had different settings and participants which may not only be a strength but due to the question of comparability, also a limitation. In two studies (Study 1 and 4b), actual coaching clients participated in a real face-to-face coaching process. However, participants of the other five studies were not actual coaching clients. They were university students who chose to participate in a coaching study. In addition, using a vignette-based approach in two studies raises the question of the applicability of our results to real-life coaching clients. However, within coaching research, it is often difficult to access real coaching clients and ask them to fill out a questionnaire. A vignette-approach where potential clients imagine participating in a coaching is of course time- and resource-saving and when researchers are cautious about the design and validity, vignettes can be an effective tool [105]. In our studies, we tried to make the situation as realistic as possible by giving participants realistic instructions. Moreover, we did not only test our hypotheses in vignette studies but also in experiments and real settings. In our experimental studies, participants set themselves actual personal goals and worked actively on the intervention, as real coaching clients would. Additionally, we commenced each coaching intervention with the setting of a personal career goal, to guarantee a certain degree of personal involvement in the subsequent intervention. Our approach is a common and worthwhile approach in psychological research that allows drawing inferences from a conveniently recruited study sample to the general population [106].

A limitation is that we mostly focused on specific aspects of the coaching process (i.e., reactions to coaching interventions concerning goal operationalization) rather than on the whole coaching process or an entire coaching session. By selectively focusing on only one factor–or intervention–influencing coaching outcome, we can derive pretty clear indications about possible origins of the observed effects. However, there are other important factors impacting upon coaching outside of the laboratory, such as interpersonal dynamics, relationship quality, or the process of goal attainment [e.g., 10, 107].

Beyond differences between the two systems, RFT makes two more important claims: First, despite personality playing a major role in determining the dominant self-regulatory strategy of an individual, a given situation can also determine which focus is dominant at any given time. Thus, the coaching context can trigger a specific regulatory focus. For example, Bozer et al. [108] found that a developmental coaching emphasizing the clients’ learning, development, and their high performance triggered a situational promotion focus while a remedial coaching emphasizing the clients’ problems, difficulties, and inadequate performance triggered a situational prevention focus. In our studies, we assessed regulatory focus using a chronic measure only. However, for studies in a specific coaching context, it is important to also assess situational regulatory focus. Additionally, promotion and prevention should not be understood as exclusive categories but rather as independent self-regulatory systems. Therefore, both promotion and prevention can be equally present in some individuals and/or situations. In fact, high levels of both orientations can have positive consequences on the self-regulatory capacities of an individual by increasing flexibility in dealing with complex tasks [109]. For better comparability, interpretability, and simplicity, we decided to use a difference score of the promotion and prevention subscales, as did previous studies [75, 76]. However, one should be aware of the independence of the two regulatory systems.

The importance of considering the situational regulatory focus as well is also evident in Scholer and Higgins’ [55] differentiation of regulatory focus on a system, strategy, and tactic level. At the chronic system level, the focus represents an immanent perspective on the world, differentiating between goals (gain/security) and anti-goals (stagnation/loss). At the situational strategy level, individuals use the preferred strategies to achieve this goal point. Here, promoters prefer promotion-oriented, eager strategies, and preventers prefer prevention-oriented, vigilant strategies. If the strategy fits the system, there is a regulatory fit. Although promoters prefer promotion-oriented strategies and preventers prefer prevention-oriented strategies, an application of these strategies is dependent on the situation in which individuals find themselves. This is the tactics level, which activates a specific focus in a very specific context. At this level, promoters can adapt their eagerness strategies and preventers their vigilant strategies to achieve the goal of the situation and even initiate opposite tactics of action. Thereby, promotion- as well as prevention- tactics require active behavior in order to approach a desired endstate [55, 110]. In Study 3a, participants in the prevention condition were asked to think about actions they should avoid, which is related to the inhibition of a behavior. However, prevention strategies and tactics needs active behaviour in order to approach security goals. Thus, a prevention-condition focusing on the actions one should perform e.g., “be careful to list very detailed actions and carefully check them”; cf. [111] would have revealed different, maybe larger effects.

Getting back to the measurement of regulatory focus, Haws et al. [112] provides a conceptual and empirical evaluation of different measures of chronic regulatory focus. They conclude that researchers should be aware of the fact that the different measurements only slightly overlap in a theoretical as well as an empirical sense and that one should carefully consider the purpose of the research when selecting a scale. In our studies, we used different regulatory focus questionnaires. In two studies (Study 3c and 4a) in which the sample consisted mainly of students, we used the Lockwood scale [113] which has been reported to be designed especially for investigating research questions in the academic setting [112]. In three studies (Study 1, 2, 3a) we used a scale developed by Sassenberg et al. [114]. The reason for developing a new scale was that the internal consistency of the prevention subscale of the RFQ [115] has often been found to be low in other languages than English. The scale capturing general regulatory focus, not aiming at a specific sample or area, showed substantial correlations with the respective scales of the RFQ [cf. 114]. Another more general scale encompassing regulatory focus in various areas of life, is the German scale developed and validated by Fellner [116]. This scale captures openness to new things and autonomy for the promotion focus and orientation to the expectations of others and sense of obligation for the prevention focus. The questionnaires seemed appropriate for our research questions. However, the coaching context might need a different focus of the questionnaire, perhaps one that more strongly emphasizes a person’s values.

Qualitatively looking at the content of the goal would also be an interesting direction for future research. A question that one could ask is whether more promotion- vs. more prevention-oriented goals affect coaching success. We performed these qualitative analyses for Study 3c (as noted by one reviewer) and found that RFI did not affect the goal formulation (see S1 File). That is, in general, most goals were more promotion-oriented (e.g., “Finding enthusiasm for my study”, “New position and gain new experience.”) rather than prevention-oriented (e.g., “Continue as before”, “Be sure I have found the right study or some other right thing to do.”). Thus, letting clients set their goals on their own (as it was the case in this study), they may be promotion-oriented most of the time. Moreover, the type of goal alone, whether promotion- or more prevention-oriented, did not affect coaching success as no significant correlations could be found. If coaches pay attention to the client’s regulatory focus and intervene in goal setting, the goals might also be prevention-oriented, depending on the client’s focus. Future research may address the question of whether the type of goal affects coaching success when it fits the client’s regulatory focus.

As for the fit effects, we tested many different dependent variables, we performed alpha corrections for all studies. Applying this correction, many of our significant results disappear calling into question our fit-hypothesis. New studies with more power that test our significant findings would provide some clarity.

### Theoretical implications

While many previous studies have provided evidence for the benefits of coaching, some studies have also highlighted the possible negative effects of coaching [117]. Further, a meta-analysis indicated that studies investigating the effectiveness of coaching yield rather heterogeneous results [15]. Shedding some light on how to minimize no or even negative outcomes and maximize positive outcomes was a major goal of the present research. Our results suggest that choosing the right coaching intervention can help coaches establish situations of motivational congruency, which subsequently facilitate successful self-regulation. Motivational congruency (or “fit”) may lead to the activation of the behavioral approach system, a motivational system triggering positive affect and motivation to initiate goal-directed behavior [118]. In contrast, choosing a non-fitting intervention can hinder the self-regulation of clients who may perceive the goal discrepancy as unmanageable and thus threatening. A further negative effect is the activation of the behavioral inhibition system, a motivational system that triggers negative affect and feelings of insecurity and anxiety [118].

Research that integrates RFT in the coaching context is scarce. However, some studies have compared solution-focused with problem-focused coaching, which can be related conceptually to comparing promotion with prevention. The findings of these studies are in line with the present line of research. Solution-focused coaching emphasizes finding solutions rather than analyzing problems, which is related to a promotion focus. Problem-focused coaching, in contrast, seeks to uncover the origins of a client’s problem, which is related to a prevention focus. Similar to outcomes in our studies for participants with a high promotion focus, solution-focused coaching seems to have advantages, such as positive affect, increased self-efficacy, and increased goal progress [21, 119, 120]. Importantly though, in some cases, solution-orientation in coaching can backfire. A study that used solution-focused coaching to reduce procrastination showed that only those individuals whose personalities resembled those of promoters benefited. Individuals who resembled preventers even reported more procrastination behavior, which could be explained by the fact that their goals did not feel self-determined but rather externally determined [26]. Additional findings revealed that problem-focused coaching processes can indeed have positive effects (reduced negative affect and increased self-efficacy) on some clients [120]. The benefits of strategies focusing explicitly on possible problems during goal pursuit have been demonstrated several times in goal-setting research. Mental contrasting and implementation intentions, for instance, are two established strategies that involve the identification of possible obstacles facing a goal and the formulation of a goal in concrete, realistic terms rather than merely as an abstract fantasy [121, 122]. Translating these findings to the context of RFT, analogies may be found to the preferred strategies of prevention-focused individuals, providing further evidence for the positive effects of identifying energy-taking aspects in life in light of possible problems in goal pursuit, as demonstrated in Study 3b.

Since the intervention itself is not the only factor influencing coaching outcomes, future studies should also focus on the role of regulatory fit in the client-coach relationship which is of fundamental importance for the coaching process. Interpersonal regulatory fit effects have already been found in situations of goal pursuit [66] and in leadership research, where followers feel more valued and leadership is perceived as more effective when their regulatory focus matches the focus or leadership style of the leader [123, 124]. We have also found interpersonal fit effects between coach and client in our Studies 4a and 4b. However, as the studies face limitations, these effects require more research.

### Practical implications

Our results have several important practical implications for coaching, counseling, and beyond. First, coaches need to develop an increased sensitivity toward the inner motives, needs, and cognitions of their clients. By being aware of the phenomenon of regulatory fit, coaches can enable self-regulation in situations where a client’s intention to change is overwhelmed by fear of failure. By flexibly adapting one’s word choice and intervention to match the perceived motivational orientation of a client, coaches foster feelings of security and openness to change. Since a person’s motives are often not observable, coaches need to refine their focus on specific keywords the client uses and in this way gather information about the present regulatory focus (promotion focus: success, change, variety, growth, opportunity, possibility, ideals, hope, desire, long-term; prevention focus: failure, security, obligations, risk, rules, duties, status-quo, uncontrollability, mistake, short-term). These keywords may be explicitly provoked, for instance, by asking clients to write down the first five associations that come to mind when they think about their goal. Additionally, increased insight into a client’s regulatory focus can be reached by integrating diagnostic instruments into the coaching process (e.g., the Lockwood scale). Further, coaches should aim at leading their clients to invest in self-reflection [125]. Learning about their own regulatory focus and the consequences of their security or growth orientation can empower clients to pursue their goals corresponding to their self-regulatory system. Self-reflection, however, should also be part of the coaches’ competencies [117]. Understanding one’s motivational drivers can help a coach assume the perspective of the other and thus determine whether an intervention is inappropriate for a particular client.

Additionally, since both motivational orientations are accompanied by advantages (e.g., promotion: optimism and openness for change, prevention: setting milestones and developing alternative plans) and disadvantages (e.g., promotion: setting goals that are hard to achieve; prevention: status quo orientation in change processes), coaches should make use of promotion and prevention advantages for the client. That is when coaching a promotion-oriented client, coaches may also explain the possible gains of prevention-oriented interventions. For example, as promoters tend to pursue goals that are difficult to achieve and sometimes abandon these as exciting new goals arise, coaches can emphasize that setting milestones is important. When coaching a prevention-oriented client and thus, focusing on security aspects, it is also important not to neglect the growth aspect. We, therefore, recommend proceeding in two phases: After identifying possible risks and obstacles, coaches should also focus on possible positive outcomes. Satisfying the security need of the client in the first step may lead to a motivational re-orientation (from security to growth) in the second step, whereby the security motive diminishes and the growth motive emerges. This could also explain findings from Study 4b, in which clients using a prevention-oriented language indicated more goal attainment when their coach used a promotion-oriented language and not when they used a prevention-oriented language.

In practice, this means that a coach should first serve the client’s most important need (growth vs. security) before confronting them with the respective other focus. Thereby, the coach can respond to individual concerns and can pick up clients where they stand. Such personalized support means that coaches support the client in their specific personal situation which includes using tools, methods, and conversational techniques that align with the client’s situation but also showing empathy with the client [36]. For example, for preventers, it is important to empathize with the negative feelings but then not to remain there in analysis and pain. Instead, a coach should then help the client to find concrete solutions and change steps. If coaches go straight into the solution without empathizing with the negative feelings, they may lose the client. If coaches get stuck in empathy, on the other hand, they may gain sympathy and closeness but they will lose the decisive progress.

When no clear dominant focus is detectable or assessing regulatory focus is impeded, it is recommended to make use of interventions integrating both communication channels simultaneously: For instance, by performing a SWOT analysis of strengths, weaknesses, opportunities, and threats or by operationalizing the goal with the identification of wish, outcome, obstacle, and plan [WOOP; 122]. As our results imply, it may also be essential to include different levels of mental abstraction in the designed interventions. Especially when coaching preventers, it is important to be mindful of their preference for concrete cognitive processes. Certainly, experienced coaches will seldom choose interventions that exclusively focus on only one regulatory focus or one construal level, since many interventions integrate both orientations to a certain extent. Nonetheless, our results suggest that clients can benefit from a conscious choice of motivationally congruent coaching interventions. Particularly when perceiving sudden resistance or motivational inhibition within a client, flexibly adapting interventions and language according to regulatory focus characteristics can be a valuable tool for every coach.

## Conclusion

Coaching represents a self-regulatory process of growth. The client, accompanied by a coach, aims to reach a desired state in the future. However, moving towards this state also means moving away from the current state. This process involves a certain degree of unpredictability and uncertainty—while some individuals perceive this process as an exciting challenge, others fear a departure from the status quo and feel confronted with a threatening situation that first needs vigilant analysis. While for promotion-focused clients, the “normal” coaching seems to work optimally, for prevention-focused clients, the coaching process should be adapted. Generally, coaching should be defined and designed as a process of exploration and growth. Nonetheless, individuals motivated by a need for security or a preference for concrete-analytical thinking should not be overlooked. Our research shows that regulatory fit is present and should not be neglected in coaching. We received small to medium effects on various relevant coaching outcomes. By integrating both foci, growth as well as security aspects, self-regulation can be facilitated and growth in turn can become possible independent of regulatory focus. Thus, even in clients with a strong prevention focus, coaches can try to establish a situation of regulatory fit, engendering the notion of feeling right and eventually unlocking the necessary doors where goal discrepancy is perceived as manageable and personal growth and behavioral change may be realized.

## Supporting information

S1 Table Measures used in the studies. (PDF)

S2 Table Means, standard deviations, and correlations in Study 3c. (DOCX)

S1 File Supporting information containing study material for Studies 2, 3c, and 4a, and further results for Study 3c. (DOCX)
