# Supplementary material for: Coaching as a growth- or security-oriented process–How regulatory fit increases coaching success
Source: PLoS One. 2023 Oct 5;18(10):e0286059. doi: 10.1371/journal.pone.0286059 (PMC10553236; doi:10.1371/journal.pone.0286059)
Supplement: S1 Table — (PDF) [file pone.0286059.s001.pdf]

Variable	Measure	Study 1	Study 2	Study 3a	Study 3b	Study 3c	Study 4a	Study 4b
**Regulatory focus**	Instrument	Sassenberg et al. [1]<br>Scale from 1 (strongly disagree) to 5 (strongly agree)<br>Dv: RFI (promotion-prevention)	Sassenberg et al. [1]<br>Scale from 1 (strongly disagree) to 7 (strongly agree)<br>Dv: RFI (promotion-prevention)	Sassenberg et al. [1]<br>Scale from 1 (strongly disagree) to 7 (strongly agree)<br>Dv: RFI (promotion-prevention)	Fellner et al. [2]<br>Scale from 1 (strongly disagree) to 5 (strongly agree)<br>Dv: RFI (promotion-prevention)	Keller and Bless [3], which is a German translation of the established General Regulatory Focus Measure [4]<br>Scale from 1 (strongly disagree) to 7 (strongly agree)<br>Dv: RFI (promotion-prevention)	Keller and Bless [3], which is a German translation of the established General Regulatory Focus Measure [4]<br>Scale from 1 (strongly disagree) to 5 (strongly agree)<br>Dv: RFI (promotion-prevention)	Promotion and prevention focus according to language analyses with PRECIRE®
	Remarks	1 item of the promotion scale and 1 item of the prevention scale were excluded due to an insufficient item-total correlation (< .25) and an increase in cronbach's alpha after exclusion.	1 item of the promotion scale and 2 items of the prevention scale were excluded due to an insufficient item-total correlation (< .25) and an increase in cronbach's alpha after exclusion.		1 item of the promotion scale was excluded due to an insufficient item-total correlation (<.25) and an increase in cronbach's alpha after exclusion.		2 items of the prevention scale were excluded due to an insufficient item-total correlation (< .25) and an increase in cronbach's alpha after exclusion.	
	Items, Examples, Cronbach's alpha	promotion: 11 items, e.g., "I am striving for success in life", α = .78<br>prevention: 7 items, e.g., "In case of important decisions, security is a core criterion that I value", α = .72	promotion: 11 items, e.g., "I am striving for success in life", α = .83<br>prevention: 6 items, "In case of important decisions, security is a core criterion that I value", α = .70	promotion: 12 items, e.g., "I am striving for success in life", α = .78<br>prevention: 8 items, "In case of important decisions, security is a core criterion that I value", α = .70	promotion: 4 items, e.g., "For the most part I solve problems in a creative manner", α = .72, prevention: 5 items, e.g., "I often think about what other people expect from me", α = .73	promotion: 9 items, e.g., "I often think about the person I would ideally like to be in the future", α = .82; prevention: 9 items, e.g., "I am anxious that I will fall short of my responsibilities and obligations.", α = .82	promotion: 9 items, e.g., "I often think about the person I would ideally like to be in the future", α = .77<br>prevention: 7 items, e.g., "I am anxious that I will fall short of my responsibilities and obligations.", α = .80	
**Satisfaction with the coach/coaching**	Instrument		Self-created<br>Scale from 1 (strongly disagree) to 7 (strongly agree).		Self-created<br>Scale from 1 (strongly disagree) to 5 (strongly agree)		Self-created<br>Scale from 1 (strongly disagree) to 5 (strongly agree)	
	Items, Examples, Cronbach's alpha		2 items, "I would benefit from this coaching", "This coaching appeals to me"		3 items, "I can imagine making use of a real-life coaching beyond this online version.", "I would recommend coaching to others.", "In my opinion, coaching can help to better achieve one's own goals.", α = .90		4 items, "I would benefit from this coach", "I would love to work on my goals with this coach", "I would enjoy a cooperation with this coach", "I like this coach" (promotion coach: α = .91; prevention coach: α = .77)	
**Trust**	Instrument						Mayer et al. [5]<br>Scale from 1 (strongly disagree) to 5 (strongly agree)<br>DV: General trust mean score (consisting of trust in ability, benevolence, integrity)	
	Remarks						We slightly adapted the items to assess people's trust in the promotion and prevention coach	
	Items, Examples, Cronbach's alpha						Ability: 2 items, "This coach provides qualified and efficient consulting", "In total, this coach appears capable and experienced to me" (promotion coach: r = .87; prevention coach: r = .72)<br>benevolence: 2 items, "This coach would go out of their way to bring me closer to my goal", "This coach would address my needs and desires" (promotion coach: r = .74; prevention coach: r = .57)<br>integrity: 2 items, I can imagine that this coach would stick to their word", "I can imagine that this coach tries to be fair in dealing with others" (promotion coach: r = .74; prevention coach: r = .82)	
**Implicit approach motivation**	Instrument	Line Bisection Task [6]<br>t1: immediately before the beginning of the first coaching session (baseline approach motivation)<br>t2: immediately after the fifth and last coaching session (final approach motivation)<br>Dv: t2-t1 (final-baseline; positive scores indicate relatively more rightward errors and thus, an increase in approach motivation)		Line Bisection Task [6]<br>t1: directly after the intervention (intervention approach motivation)<br>t2: after the questionnaires (final approach motivation)<br>Dv: t2-t1 (final-intervention; positive scores indicate relatively more rightward errors and thus, approach motivation)		Line Bisection Task [6]<br>t1: Beginning of the study (baseline approach motivation)<br>t2: Directly after the intervention (intervention approach motivation)<br>Dv: t2-t1 (intervention-baseline; positive scores indicate relatively more rightward errors and thus, approach motivation)		

	Instruction for participants	"On this page you see some horizontal lines. Please separate each line into two equal parts by a short, single stroke. Draw a line through the middle of each line without measuring or folding."		"Below you can see some horizontal lines. Now please mark the center of each line with the cursor. Follow your intuition without thinking too long."		t1: Baseline approach motivation: "Below you can see some horizontal lines. Now please mark the center of each line with the cursor. Follow your intuition without thinking too long."<br>t2: Intervention approach motivation: "Please continue thinking about your goal. Below you can see some horizontal lines. Now please mark the center of each line with the cursor. Follow your intuition without thinking too long."		
	Items, Examples, Cronbach's alpha	t1: Baseline approach motivation: 10 lines, α = .75<br>t2: Final approach motivation: 10 lines, α = .70		t1: Intervention approach motivation: 10 lines, α = .96<br>t2: Final approach motivation: 10 lines, α = .97		t1: Baseline approach motivation: 10 lines, α = .86<br>t2: Intervention approach motivation: 10 lines, α = .68		
**Handedness**	Instrument	1 item asking whether they were right-handed, left-handed, or ambidextrous		Edinburgh Handedness Inventory (7)<br>scale: 1 (left), 2 (right), 3 (both)<br>Dv: Percentage handedness (Sum right - Sum left) /10 * 100 (positive values indicate stronger right-handedness		Edinburgh Handedness Inventory (7)<br>scale: 1 (left), 2 (right), 3 (both)<br>Dv: Percentage handedness (Sum right - Sum left) /10 * 100 (positive values indicate stronger right-handedness		
	Instruction for participants			"Please indicate which hand you prefer to use when performing the following activities"		"Please indicate which hand you prefer to use when performing the following activities"		
	Items, Examples, Cronbach's alpha			8 items, e.g. "writing", M = 76.92, SD = 54.12		9 items, e.g. "writing", M = 69.96, SD = 43.56		
**Self-reported approach motivation**	Instrument	High approach state [8], item "determined" [9]<br>Scale from 1 (not at all) to 5 (very much).	Item "determined" [29]<br>Scale from 1 (strongly disagree) to 7 (strongly agree).					
	Instruction for participants	Please indicate to what extent the following statements apply to you.						
	Items, Examples, Cronbach's alpha	Self-reported approach motivation: 4 items, "Right now, after the coaching session, I feel… [energized, powerful, capable, goal-focused], a = .85<br>Self-reported approach motivation "determined" - 1 item, determined	Self-reported approach motivation "determined": "I am determined to participate in this coaching"					
**Self-efficacy**	Instrument			Jerusalem & Schwarzer [10]<br>Scale from 1 (strongly disagree) to 7 (strongly agree)	Jerusalem & Schwarzer [10]<br>Scale from 1 (disagree) to 4 (agree)<br>dV: Change in self-efficacy: t2-t1, t3-t1	ASKU [11] based on Jerusalem & Schwarzer [10]<br>Now think about your studies or your profession. To what extent do the following statements in this area apply to you?<br>Scale from 1 (strongly disagree) to 7 (strongly agree)		
	Items, Examples, Cronbach's alpha			10 items, e.g., "When I am confronted with a problem, I can usually find several solutions", α = .89	10 items, e.g., "When I am confronted with a problem, I can usually find several solutions", αt1 = .82, αt2 = .88, αt3 = .84;	3 items; e.g., "I can rely on my own abilities in difficult situations", α = .85		
**Goal-related motivation**	Instrument			Situational Motivation Scale [SIMS; 12]<br>Scale from 1 (strongly disagree) to 7 (strongly agree)		Situational Motivation Scale [SIMS; 12]<br>"Now think of activities you perform in relation to your goal. Please think about why you are doing this activity. "<br>Scale from 1 (strongly disagree) to 7 (strongly agree)		
	Items, Examples, Cronbach's alpha			Intrinsic motivation regarding goal-related activities: 4 items; e.g., "Because I feel good when doing this activity", α = .88;<br>Identified regulation regarding goal-related activities: 4 items; e.g., "because I am doing it for my own good", α = .71		Intrinsic motivation regarding goal-related activities: 4 items; e.g., "Because I feel good when doing this activity", α = .89;<br>Identified regulation regarding goal-related activities: 4 items; e.g., "because I am doing it for my own good", α = .81		
**Goal commitment**	Instrument			German version (13) of Klein et al. (14)<br>Scale from 1 (strongly disagree) to 7 (strongly agree)		German version (13) of Klein et al. (14)<br>Scale from 1 (strongly disagree) to 7 (strongly agree)		
	Items, Examples, Cronbach's alpha			5 items, e.g., "I am strongly committed to pursuing this goal", α = .65		5 items, e.g., "I am strongly committed to pursuing this goal", α = .68		
**Self-esteem**	Instrument				Rudolph et al. [15]<br>Scale from 1 (strongly disagree) to 5 (strongly agree)<br>dV: Change in self-esteem: t2-t1, t3-t1			
	Items, Examples, Cronbach's alpha				15-items, e.g., "I feel good about myself", αt1 = .86, αt2 = .87, αt3 = .87			

**Affect**	Instrument				Positive and Negative Affect Schedule [PANAS; 16]<br>"How do you feel right now?"<br>Scale from 1 (strongly disagree) to 5 (strongly agree)<br>Dv: Change in affect: Positive-negative affect for the three measurement times (t2-t1, t3-t1)			
	Items, Examples, Cronbach's alpha				Positive affect: 5 items, e.g., "determined", "inspired", αt1 = .89, αt2 = .92, αt3 = .92;<br>Negative affect: 10 items; e.g., "distressed", "nervous", αt1 = .79, αt2 = .80, αt3 = .86			
**Goal attainment**	Instrument	Self-created<br>Scale from 1 (not at all) to 10 (fully)				Self-created<br>Scale from 1 (not at all) to 10 (fully)		Self-created<br>Scale from 0 (unfavorable result) to 4 (best possible result)
	Items, Examples, Cronbach's alpha	Target goal attainment: ""On a scale from 1 (not at all) to 10 (fully), where would you like to be after the coaching with your goal attainment?"<br>Final goal attainment: "So far, to what extent have you attained your goal on a scale from 1 (not at all) to 10 (fully)?"<br>Goal discrepancy score: Final goal attainment-target goal attainment				1 item: "So far, to what extent have you attained your goal on a scale from 1 (not at all) to 10 (fully)		Before the coaching session, clients wrote down up to three coaching goals for the following month and indicated their importance from 0.50 (low importance) to 1.00 (very important). One month after the coaching, clients reflected on these goals and indicated their attainment on a scale from 0 (unfavorable result) to 4 (best possible result). As most clients only wrote down one goal, we performed our analyses with this one goal. We built a goal attainment index indicating people's goal attainment weighted by importance.
**Value from fit**	Instrument		Self-created following Latimer [17] and literature on feeling right [e.g., 18]<br>Scale from 1 (strongly disagree) to 7 (strongly agree)			Self-created following Latimer [17]<br>Scale from 1 (strongly disagree) to 7 (strongly agree)		
	Items, Examples, Cronbach's alpha		6 items, "Participating in this coaching will give me joy", "I'm motivated to participate in this coaching", "It will feel good to participate in this coaching", "Once I have participated in the coaching, I will feel positive about it", "The participation in the coaching is valuable to me", "It feels right to participate in this coaching"; Cronbach's alpha for the six different coaching offers ranged between α = .87 and α = .96			5 items, "Participating in this coaching will give me joy", "I'm motivated to participate in this coaching", "It will feel good to participate in this coaching", "Once I have participated in the coaching, I will feel positive about it", "The participation in the coaching is valuable to me"; α = .84		
**Intended time of goal initiation**	Instrument					Self-created		
	Items, Examples, Cronbach's alpha					When will you start realizing your goal?" Scale ranging from 1 (today) to 5 (in 1 month)<br>As a statistical control for the intended time of goal initiation, we used the item: "When do you want to have reached your goal?" on a scale ranging from 1 (in 1–2 weeks) to 6 (in 4 months)		
**Coaching progress**	Instrument							Self-created [cf. 19]<br>Scale from 1 (strongly disagree) to 7 (strongly agree).
	Items, Examples, Cronbach's alpha							Enhanced understanding: 3 items, e.g., "I have gained new perspectives on challenging situations today.", α = .72<br>Strengthened motivation: 3 items, e.g., "I now know more precisely what I want to achieve", α = .59<br>Facilitated implementation: 4 items, e.g., "I will now use my strengths more successfully.", α = .81

References

1. Sassenberg K, Ellemers N, Scheepers D. The attraction of social power: The influence of construing power as opportunity versus responsibility. J Exp Soc Psychol [Internet]. 2012;48(2):550–5. Available from: http://dx.doi.org/10.1016/j.jesp.2011.11.008
2. Fellner B, Holler M, Kirchler E, Schabmann A. Regulatory Focus Scale (RFS): Development of a scale to record dispositional regulatory focus. Swiss J Psychol [Internet]. 2007;66(2):109–16. Available from: https://psycnet.apa.org/fulltext/2007-09625-004.pdf
3. Keller J, Bless H. Regulatory fit and cognitive performance: the interactive effect of chronic and situationally induced self-regulatory mechanisms on test performance. Eur J Soc Psychol [Internet]. 2006;36(3):393–405. Available from: http://dx.doi.org/10.1002/ejsp.307
4. Lockwood P, Jordan CH, Kunda Z. Motivation by positive or negative role models: regulatory focus determines who will best inspire us. J Pers Soc Psychol [Internet]. 2002;83(4):854–64. Available from: http://dx.doi.org/10.1037//0022-3514.83.4.854
5. Mayer RC, Davis JH, Schoorman FD. An integrative model of organizational trust. Acad Manag Rev. 1995;20(3):709-734. Available from: https://doi.org/10.5465/amr.1995.9508080335.
6. Jewell G, McCourt ME. Pseudoneglect: a review and meta-analysis of performance factors in line bisection tasks. Neuropsychologia [Internet]. 2000;38(1):93–110. Available from: http://dx.doi.org/10.1016/s0028-3932(99)00045-7
7. Oldfield RC. The assessment and analysis of handedness: the Edinburgh inventory. Neuropsychologia. 1971;9(1):97-113. doi: 10.1016/0028-3932(71)90067-4.
8. Greenaway KH, Storrs KR, Philipp MC, Louis WR, Hornsey MJ, Vohs KD. Loss of control stimulates approach motivation. J Exp Soc Psychol [Internet]. 2015;56:235–41. Available from: http://dx.doi.org/10.1016/j.jesp.2014.10.009

9. Harmon-Jones C, Schmeichel BJ, Mennitt E, Harmon-Jones E. The expression of determination: similarities between anger and approach-related positive affect. J Pers Soc Psychol [Internet]. 2011;100(1):172–81.
10. Jerusalem M, Schwarzer R. Skalen zur Erfassung von Lehrer-und Schülermerkmalen. Dokumentation der psychometrischen Verfahren im Rahmen der Wissenschaftlichen Begleitung des Modellversuchs Selbstwirksame Schulen. Berlin: Freie Universität Berlin; 1999.
11. Beierlein C, Kovaleva A, Kemper CJ, Rammstedt B. Ein Messinstrument zur Erfassung subjektiver Kompetenzerwartungen: Allgemeine Selbstwirksamkeit Kurzskala (ASKU). Methoden, Daten, Analysen (mda). 2013;7(2):251-278.
12. Guay F, Vallerand RJ, Blanchard C. On the assessment of situational intrinsic and extrinsic motivation: The Situational Motivation Scale (SIMS). Motiv Emot. 2000;24(3):175-213. doi: 10.1023/A:1005614228250.
13. Storch M. Motto-Ziele, S.M.A.R.T.-Ziele und Motivation. In: Birgmeier B, editor. Coachingwissen. Ansätze, Betrachtungen, Konzepte und Entwürfe zur Theorie- und Wissenschaftsorientierung im Coaching. Wiesbaden: VS-Verlag; 2009. p. 183-206. doi: 10.1007/978-3-531-91766-5_12.
14. Klein HJ, Wesson MJ, Hollenbeck JR, Wright PM, DeShon RP. The assessment of goal commitment: A measurement model meta-analysis. Organ Behav Hum Decis Process. 2001;85(1):32-55. doi: 10.1006/obhd.2000.2931.
15. Rudolph A, Schütz A, Schröder-Abé M. Validation of a German version of the state self-esteem scale. Poster presented at: 29th International Congress of Psychology; 2008 July; Berlin.
16. Krohne HW, Egloff B, Kohlmann CW, Tausch A. Untersuchungen mit einer deutschen Version der "Positive and Negative Affect Schedule" (PANAS). Diagnostica. 1996;42(2):139-156. doi: 10.1037/t49650-000.
17. Latimer AE, Rivers SE, Rench TA, Katulak NA, Hicks A, Hodorowski JK, et al. A field experiment testing the utility of regulatory fit messages for promoting physical activity. J Exp Soc Psychol [Internet]. 2008;44(3):826–32. Available from: http://dx.doi.org/10.1016/j.jesp.2007.07.013
18. Camacho CJ, Higgins ET, Luger L. Moral value transfer from regulatory fit: What feels right is right and what feels wrong is wrong. J Pers Soc Psychol [Internet]. 2003;84(3):498–510. Available from: http://dx.doi.org/10.1037/0022-3514.84.3.498
19. Behrendt P, Mühlberger C, Göritz AS, Jonas E. Relationship, purpose, and change—An integrative model of coach behavior. Consult Psychol J [Internet]. 2021;73(2):103–21. Available from: http://dx.doi.org/10.1037/cpb0000197
