# Supplementary material for: Coaching as a growth- or security-oriented process–How regulatory fit increases coaching success
Source: PLoS One. 2023 Oct 5;18(10):e0286059. doi: 10.1371/journal.pone.0286059 (PMC10553236; doi:10.1371/journal.pone.0286059)
Supplement: S2 Table — (DOCX) [file pone.0286059.s002.docx]

# Study 3c

**S2 Table. Means, standard deviations, and correlations in Study 3c.**

| **Variable** | ***M*** | ***SD*** | **1** | **2** | **3** | **4** | **5** | **6** | **7** | **8** | **9** | **10** |
| --- | --- | --- | --- | --- | --- | --- | --- | --- | --- | --- | --- | --- |
| 1. RFI (Promotion-Prevention) | 0.93 | 1.27 | - |  |  |  |  |  |  |  |  |  |
| 2. Promotion | 5.17 | 0.86 | .60** | - |  |  |  |  |  |  |  |  |
| 3. Prevention | 4.25 | 1.02 | -.74** | .10 | - |  |  |  |  |  |  |  |
| 4. Value from fit | 6.16 | 0.77 | .29^**^ | .32** | .09 | - |  |  |  |  |  |  |
| 5. Goal attainment | 4.08 | 2.35 | .12 | .06 | .10 | -.08 | - |  |  |  |  |  |
| 6. Self-efficacy | 5.46 | 0.89 | .44^**^ | .16* | -.41** | .20** | .15* | - |  |  |  |  |
| 7. Intrinsic motivation | 4.83 | 1.44 | .19** | .05 | -.20** | .24** | .17* | .22** | - |  |  |  |
| 8. Identified regulation | 5.68 | 1.13 | .21** | .17* | -.12 | .34** | .14(*) | .18* | .72** | - |  |  |
| 9. Goal commitment | 6.09 | 0.79 | .17* | .19** | -.05 | .46** | .06 | .22** | .14(*) | .29** | - |  |
| 10. Intended time of goal initiation | 1.94 | 1.08 | -.06 | -.14 | -.05 | -.15* | -.19* | .05 | -.14(*) | -.18* | -.10 | - |

(*) *p* < .10; * *p* < .05; ** *p* < .01
