# Supplementary material for: Coaching as a growth- or security-oriented process–How regulatory fit increases coaching success
Source: PLoS One. 2023 Oct 5;18(10):e0286059. doi: 10.1371/journal.pone.0286059 (PMC10553236; doi:10.1371/journal.pone.0286059)
Supplement: S1 File — (DOCX) [file pone.0286059.s003.docx]

# Study 2

## Study material

*Promotion-focused* coaching:

As a future manager, do you want to act successfully to realize your wishes and ideals in this position? In this coaching, we explore the opportunities of your new position and look at possibilities for change that will promote your personal development and present you with exciting challenges. This will enable you to increase your success as a manager and to grow beyond yourself in your new role.

*Prevention-focused* coaching:

As a future manager, do you want to avoid beginner's mistakes to reliably complete your tasks and duties in this position? In this coaching, we analyze potential risks and develop step-by-step concrete plans to prevent such risks and avoid mistakes. This way you will meet your responsibility as a conscientious manager and experience the necessary security in the everyday life of your new role.

*Neutral* coaching*:*

You will take over a leadership position in the future? The coaching prepares you for this position in the best possible way with suitable exercises for self-reflection. This will enable you to perform your role as a leader accurately.

# Study 3c

## Study material

*Why task*:

Why do I want to achieve what I have set out to do? This exercise will help you increase your awareness of why you want to achieve your goal. In this way, you will develop an awareness of the real reason for your goal. For everything we do in life, there is always a reason why and for what we do it. The reasons for our behavior are closely related to our overall goals. Our concrete behavior can usually be explained by our superordinate goals.

Why do you want to achieve your formulated goal? Please work, as in the example, from top to bottom. The important thing here is that you edit each of the five fields.

1. Please note again your goal: ____________________________
2. Why do you want to do this or achieve this? Because...: __________________________
3. Why do you want to do this or achieve this? Because...: __________________________
4. Why do you want to do this or achieve this? Because...: ____________________
5. Why do you want to do this or achieve this? Because...:

*How task*:

How will I achieve what I have set out to do? This exercise will help you increase your awareness of exactly how you will achieve your goal. So you will develop an awareness of the process on the way to your goal. For everything we do in life, there is a process to how we do it. We can often trace our overall goals back to our specific behavior.

What are the concrete steps you will take to achieve your formulated goal? Please work, as in the example, from top to bottom. The important thing here is that you edit each of the five fields.

1. Please note again your goal: ____________________________
2. How will you do this or achieve this? By...: __________________________
3. How will you do this or achieve this? By...: __________________________
4. How will you do this or achieve this? By...: _________________________
5. How will you do this or achieve this? By...: _________________________

## Further results – qualitative analyses of the goals

To examine whether the type of goal produced contributes to the finding that preventers showed positive effects when they worked on the how task and negative effects when they worked on the why task, we qualitatively analyzed the content of the goals that participants wrote down. Two people rated the goals according to being more prevention- or promotion-oriented on a scale from -2 = prevention, -1 = rather prevention, 0 = neutral or both, +1 = rather promotion, 2 = promotion. Interrater-reliability (ICC estimates) and their 95% confidence intervals were calculated using SPSS based on a mean-rating, consistency, and a two-way mixed-effects model. Results showed a good agreement between the raters, ICC (2, 186) = 0.79, p < 0.001, 95% CI [0.72, 0.84] (Koo and Li, 2016), so we built a mean score of the goal ratings (*M* = .63, *SD* = .88). The mean shows that the goals were rated as being more promotion- than prevention-oriented.

To test whether chronic regulatory focus is associated with more promotion- or prevention-oriented goals and whether the type of goal is associated with coaching success, we performed correlations. The correlation between RFI and the mean goal rating shows no significant correlation, *r* = -.01, *p* = .925. Looking at the separate promotion/prevention subscales, the results show a significant positive correlation of promotion with the goal rating, *r* = .15, *p* = .043, i.e. the more promotion, the more promotion-oriented goals people build. Although the correlation between prevention and the goal rating failed to reach the sign. level of 5%, it was in the positive direction, *r* = .13, *p* = .071. Thus, it might be the case that in coaching, promotion- as well as prevention-oriented individuals set themselves rather promotion- than prevention-oriented goals in coaching.

The correlations of the mean goal rating with the dependent variables were non-significant, indicating that the type of goal, whether more promotion- or more prevention-oriented, was not associated with coaching success.

To examine whether the type of goal together with RFI or the intervention produces fit effects, we performed two-way interactions. We used model 2 of the SPSS macro PROCESS by Hayes (67) with the coaching intervention (dummy coded: concrete task = 0, abstract task = 1) as an independent variable, RFI and goal rating as moderator variables, and implicit approach motivation, goal attainment, self-efficacy, intrinsic motivation, identified regulation, goal commitment, and intended time of goal initiation as dependent variables. We found a significant two-way interaction for individuals’ intended time of goal initiation, b = 0.13, SE = .07, 95% CI [0.03; 0.26], *t*(183) = 2.01, *p* =. 046, indicating that promoters who set themselves promotion-oriented goals take longer to start realizing their goals than promoters who set themselves prevention-oriented goals, *p* = .020. All other results were non-significant.

# Study 4a

## Study material

The *promotion coach* was described as follows:

Mr./Mrs. M. has been working as a successful coach for many years and is characterized by a creative and curious way of working, in which he/she always tries out new and unconventional coaching methods. The coach has the ability to inspire and motivate his/her clients to realize their own potential and to grow beyond themselves. In order to achieve his/her own professional goals, Mr./Mrs. M. is oriented towards his/her personal ideals, wishes and hopes and is always looking for new exciting challenges for himself/herself in order to grow from them and develop personally.

The *prevention coach* was described as follows:

Mr./Mrs. K. has been working as a successful coach for many years and is characterized by a reliable and conscientious way of working and uses established and recognized coaching methods. The coach shows a high sense of responsibility towards his/her clients. The coach sees it as his/her task in particular to address the clients’ concerns and fears that accompany the coaching goal. In order to avoid unnecessary risks, Mr./Mrs. K. prefers to take the safe route in the coaching profession. He/she succeeds particularly well in this by always keeping own professional tasks and duties in mind and setting goals based on this.
